# Magnetoinductive waves in attenuating media

**DOI:** 10.1038/s41598-021-85838-7

**Published:** 2021-04-07

**Authors:** Son Chu, Mark S. Luloff, Jiaruo Yan, Pavel Petrov, Christopher J. Stevens, Ekaterina Shamonina

**Affiliations:** 1 Department of Engineering Science, University of Oxford, Oxford, OX1 3PJ UK; 2 Canadian Nuclear Laboratories, Chalk River, ON K0J 1J0 Canada; 3 Department of Physics and Astronomy, University of Exeter, Exeter, EX4 4QL UK

**Keywords:** Electrical and electronic engineering, Electronic and spintronic devices

## Abstract

The capability of magnetic induction to transmit signals in attenuating environments has recently gained significant research interest. The wave aspect—magnetoinductive (MI) waves—has been proposed for numerous applications in RF-challenging environments, such as underground/underwater wireless networks, body area networks, and in-vivo medical diagnosis and treatment applications, to name but a few, where conventional electromagnetic waves have a number of limitations, most notably losses. To date, the effects of eddy currents inside the dissipative medium have not been characterised analytically. Here we propose a comprehensive circuit model of coupled resonators in a homogeneous dissipative medium, that takes into account all the electromagnetic effects of eddy currents, and, thereby, derive a general dispersion equation for the MI waves. We also report laboratory experiments to confirm our findings. Our work will serve as a fundamental model for design and analysis of every system employing MI waves or more generally, magnetically-coupled circuits in attenuating media.

Faraday’s law of magnetic induction is the fundamental principles of countless technological applications—from electrical generators, transformers, inductors^1^ to magnetic resonance imaging^2^, induction logging^3^ and modern wireless power transfer^4^. Inspired by the invention of metamaterials^5–7^, magnetoinductive (MI) waves were first observed within a chain of magnetic resonant circuits^8,9^, and owe their existence to the near field coupling between those elements. MI waves exhibit several exciting advantages that may be exploited for potential applications, such as the simplicity of design, the relatively simple mathematical description of their operation, the full-dimensional penetration (existing in the form of 1-D^8–10^, 2-D^9,11,12^ and 3-D waveguide structures^9,13^), and the wide range of operating frequency, from the MHz to the THz region^8–14^. In the last decade, MI waves have gained significant attention as an emerging and favourable communications approach for non-ideal transmission environments^15–20^. They were first proposed in underground wireless networks^15,17^ and then adapted in underwater communications over the conventional acoustic, optical, and electromagnetic techniques^18,19^. Apart from the field of underground/underwater communications, the study of coupled resonant circuits in a dissipative medium has also attracted great interest in medical diagnosis and treatment applications^20–22^ because human organs and tissues are moderately conductive at radio frequencies^23–25^.

In contrast to free space, in an attenuating environment a time varying magnetic field induces eddy currents throughout the medium, which inevitably attenuates the inter-element coupling strength due to Lenz’s law. In most current literature, considering only the path loss suffices to describe the effect of eddy currents^16–19^. Particularly, the attenuation of the near fields due to the dissipative medium has been primarily accounted by a skin-depth-based-plane-wave model^26,27^, where the mutual coupling is assumed to undergo the same decay rate as plane waves propagating in the lossy medium. Recent work^28^ has explored the validity of that model via simulations and experiments, showing that it is too simplistic and crude. By adopting the mathematical approach in^28^, the authors in^29^ developed a model to calculate the mutual inductance between two coils, completely immersed in an infinite homogeneous conductive medium, e.g., buried in the ground or submerged in the water. They showed that the mutual coupling is generally complex and frequency dependent due to the additional contributions from eddy currents. In addition, the eddy currents induced in a non-ideal medium are expected to alter the self impedance of the radiators as the absorption of electromagnetic waves for near-field systems generally creates a feedback effect on the load of the transmitter. Recently, advanced studies^30,31^ in wireless power transfer for underwater vehicles have investigated the loss and the detuning effects due to eddy currents in seawater. Although these works proposed an analytical method to quantitatively evaluate the eddy current impacts, some important parameters are obtained through simulation and experimental means^30,31^.

In this paper, we present a general equivalent circuit model for two coupled circuits in infinite homogeneous dissipative media as a combination of circuit theory and field theory. Very different from the previously reported models, our work analytically incorporates all electromagnetic coupling and feedback effects. We further derive and experimentally demonstrate the dispersion relationship of MI waves in a non-ideal medium with variable conductivity.

## Results

### Equivalent circuit model

**Figure 1:**
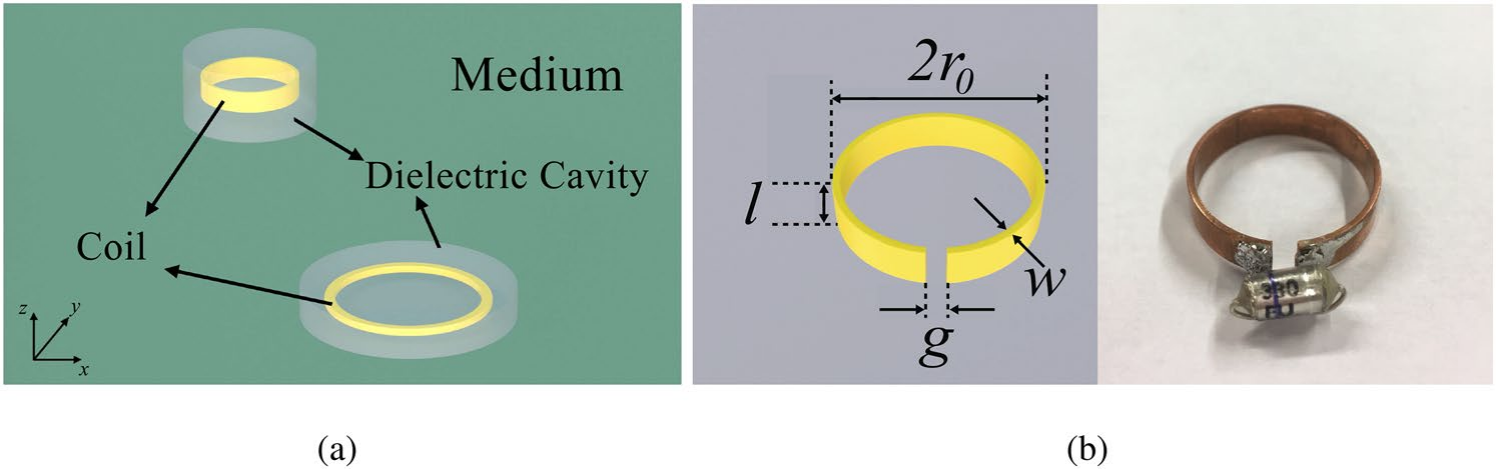
(**a**) Schematic diagram of two coupled coils insulated by dielectric cavities and completely immersed inside an infinite homogeneous dissipative medium. (**b**) Diagram (left) and photograph (right) of the coils. Each coil is a split ring resonator, the geometry of which is determined by mean radius $r_0=11$ mm, length $l=5$ mm, width $w=1$ mm, and gap width $g=1$ mm.

We start by considering a general configuration of two coupled coils with the same orientation placed in lossless dielectric cavities ($\sigma =0$) in an infinite homogeneous dissipative environment ($\sigma _m\ne 0$), as shown in Fig. 1a. The purpose of the insulators is to prevent direct conduction currents from the transmitter and current leakage to the medium background. The time-varying magnetic fields generated by circulating electric currents in the two coils induce eddy currents in the medium nearby due to Faraday’s law of induction. The magnetic interactions between those currents and the induced eddy currents modify the self impedances of the coils ($Z_n$) as well as the mutual inductance between them ($M_0$). We propose two Kirchhoff terms—complex self inductances ($\Delta {{\mathcal {L}}}$) and complex mutual inductance (${{\mathcal {M}}}$)—to describe these effects of the eddy currents respectively. Applying the Kirchhoff’s voltage law, the general equivalent circuit model of this system is expressed by:

1 $$\begin{aligned} \begin{bmatrix} V_1 \\ V_2 \end{bmatrix} = \begin{bmatrix} Z_1+j\omega \Delta {{\mathcal {L}}}_1 &{} j\omega {{\mathcal {M}}} \\ j\omega {{\mathcal {M}}} &{} Z_2+j\omega \Delta {{\mathcal {L}}}_2 \end{bmatrix} \begin{bmatrix} I_1 \\ I_2 \end{bmatrix} \end{aligned}$$

where the subscript (1, 2) denotes the coil number, $Z_n$ is the self impedance of the coil *n* in the absence of conducting surrounding, $V_n$ and $I_n$ are the applied voltages and current responses of the coil *n* respectively, and $\omega$ is the angular frequency. The complex self inductances $\Delta {{\mathcal {L}}}$ represents the coupling of a coil and the eddy currents it generates whereas the complex mutual inductance is the summation of the mutual inductance in free space ($M_0$), and the coupling of a coil and the eddy currents generated by a nearby coil ($M_E$), i.e., ${{\mathcal {M}}}=M_0+M_E$. When the medium is vacuum, it has no impact on the magnetic channels and hence, all the additional coupling—the complex self inductance and the added mutual inductance—no longer exist, i.e., $\Delta {{\mathcal {L}}}_1=\Delta {\mathcal {L}}_2=M_E=0$. Consequently, (1) reduces to the circuit model of two coils coupled in free space.

To calculate the complex coefficients, we first formulate the differential equation for the magnetic vector potential under the assumption that the dielectric cavities are replaced by infinitely wide dielectric layers (see Supplementary Note 1A). The complex self and mutual inductance can be calculated from the eddy-current-based fields. We then perform the full-wave electromagnetic simulations in CST EM studio (CST EMS) (see Methods) in order to consider the cases when the dielectric cavities are finite. Since one of the potential applications of MI waves is in magnetic resonance imaging, the resonance frequency is set to $f_0 = 46$ MHz, equivalent to hydrogen MRI in 1.08 T magnetic field. Assuming each coil is a split ring resonator made of annealed copper where the dimensions are: mean radius $r_0=11$ mm, length $l=5$ mm, width $w=1$ mm, and gap width $g=1$ mm, see Fig. 1b. Also, we assume that resistance of the coil is mainly due to the skin effect. The circuit parameters are calculated by using standard formulas for loop antennas^32^ as follows: $R_0 = 10.19$ m$\Omega$, $L_0 = 30.46$ nH, $C_0 = 393$ pF, and $Q= 432$. In the simulations, we vary the size of the cavities to justify our assumption of infinitely wide dielectric layers. It is clear that the larger the insulators are, the more accurate the analytical method provides. For the purpose of comparison, we present the simulation results corresponding to three different insulator radii of $R = 1.1r_0$, $2r_0$, and $3r_0$.

**Figure 2:**
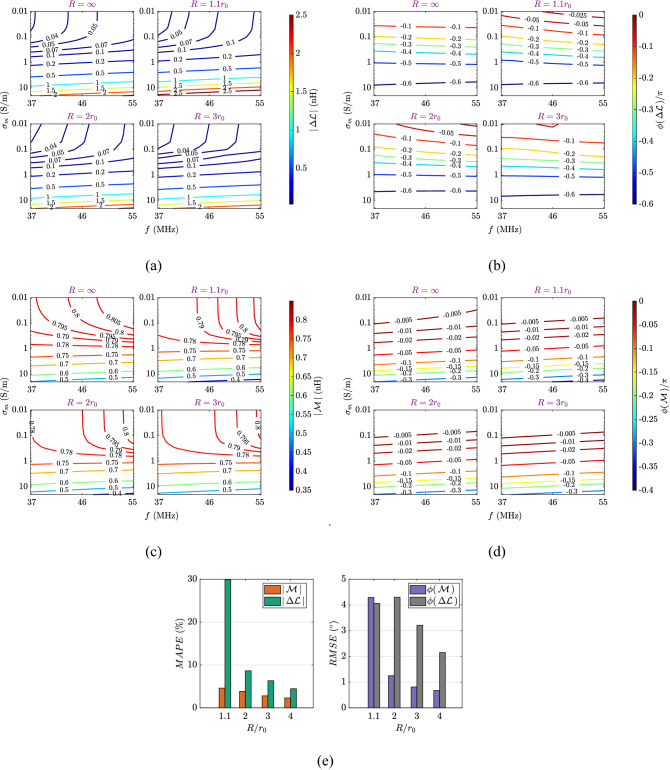
Complex Kirchhoff coefficients. (**a**–**d**) Modulus and phase of complex Kirchhoff coefficients $\Delta {\mathcal {L}}$ and ${\mathcal {M}}$ as a surface function of the working frequency and the medium conductivity. The medium conductivity is from $\sigma _m= 0.01$ to $\sigma _m = 20$ S/m while the working frequency ranges between $f = 37$ to $f = 55$ MHz. The vertical distance between the two coils is 30 mm while horizontal displacement is 0 mm. In each sub-figure, (Top left) the analytical values ($R=\infty$) are compared to CST EMS simulation results for (Top right) $R = 1.1r_0$, (Bottom left) $R = 2r_0$, and (Bottom right) $R = 3r_0$. (**e**) (left) Mean absolute percentage error (in percent) for the modulus of the complex Kirchhoff coefficients calculated by the analytical solutions with respect to CST EMS simulation results, and (right) root mean square error (in arcdegree) for the phase of the complex Kirchhoff coefficients calculated by the analytical solutions with respect to CST EMS simulation results.

Firstly, the theoretical predictions for the complex Kirchhoff coefficients are compared to those obtained with CST EMS over a range of frequency, *f* (from 37 to 55 MHz), and medium conductivity, $\sigma _m$ (from 0.01 to 20 S/m where the conductivity of most soils and biological matters is found^23–25,33^). The two indentical coils are placed coaxially at a distance of 30 mm from each other. Unlike normal inductive terms which are always considered constant at low frequency, the complex coefficients are indeed frequency-dependent, as shown in Fig. 2a–d. The magnitude of the real and imaginary parts of the complex self inductance is directly proportional to the working frequency (see Fig. S2 in the Supplementary Information) due to the stronger eddy current effects at higher frequencies. We note that the complex self inductance always has a negative imaginary part, representing additional losses due to the conductive medium, as shown in Fig. S2b. The real part of the complex self inductance can be either sign depending on the nature of the environment, representing the change in self-flux of the circuit. When the medium is highly permittive but weakly conductive (lossy dielectric material for an example), the real part of the complex self inductance is positive due to the flux reinforcement of the displacement eddy currents, see Supplementory Information Fig. S2a. As the conductivity increases, the eddy currents induce an opposite EMF back to the transmitting coil as expected from Lenz’s law so that the complex self inductance’s real part is negative, representing the reduction in self-flux, as shown in Supplementory Information Fig. S2a. In any case, the surrounding medium probably alters the resonant frequency of the coils. Additionally, stronger eddy currents at higher frequencies and larger values of conductivity attenuate the magnetic flux towards the other coil as well as introduce a phase shift in the magnetic field that leads to a complex mutual inductance between the two coils, see Fig. S2c,d and results in^28,29^.

Next, we examine the validity of the analytical model by comparing the theoretical values of the complex coefficients to those obtained with CST EMS for different cavity sizes (Fig. 2e). When the radius of the cavities is three times larger than the coil radius $R\ge 3 r_0$, we find excellent agreement between the analytical model and the numerical simulations with a mean absolute percentage error less than $7\%$ for the modulus and a root mean square error less than 4$^\circ$ for the phase, over the simulated ranges of conductivity and working frequency. More details on the comparison for variable vertical distance and horizontal displacement can be found in Supplementary Information Note 1B.

### Network analysis

**Figure 3:**
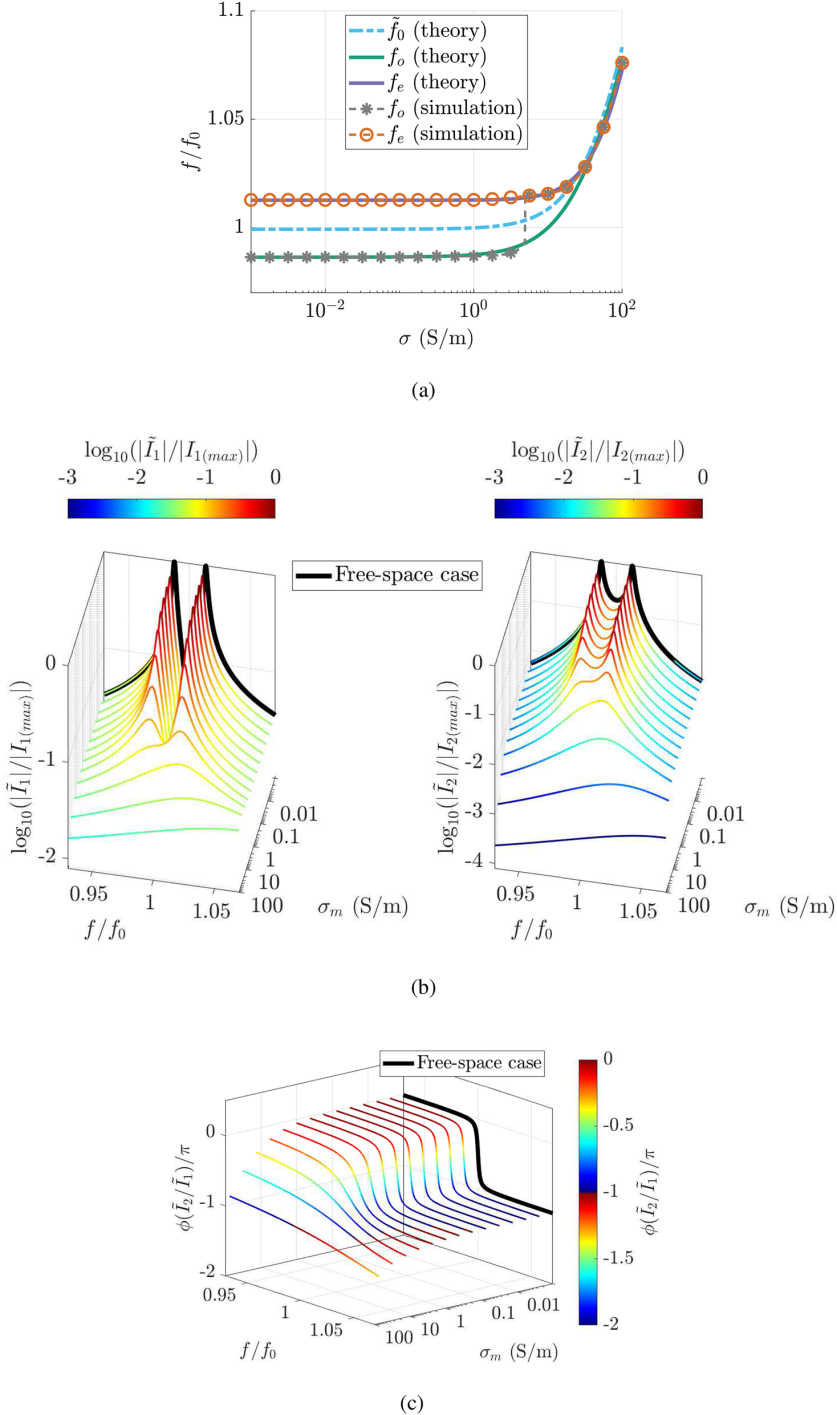
Network analysis. (**a**) Impact of medium’s conductivity on the resonant frequency of an individual resonator and the frequency splitting of two coupled identical resonators. (**b**) 3-D waterfall map of normalised magnitude of the current distribution versus frequency and medium conductivity in (left) the transmitter and (right) the receiver. (**c**) 3-D waterfall map of phase difference between the currents in the transmitter and the receiver versus frequency and medium conductivity. The medium has relative permittivity of $\epsilon _r = 78$, relative permeability of $\mu _r = 1$, and conductivity from $\sigma =0.01$ to $\sigma =100$ S/m. For the purpose of comparison, current responses of the two coils in the free space case are included as a black line. The current moduli are normalised to the maximum value of the corresponding currents in the free space while the current phases are normalised to $\pi$.

With the equivalent circuit model of two coupled resonators being modified significantly because of the additional coupling terms, we also perform detailed network analysis. Having proved the merit of the novel analytical solutions with an excellent accuracy not exceeding 7% in terms of the mean absolute percentage error with respect to the CST simulated results for reasonable values of the parameters, only results from those expressions are considered in this part. Apart from the resonant frequency and Q-factor (see Supplementary Information Note 2A), another main interest for every coupled resonant system is the frequency splitting phenomenon. In the strongly-coupled regime, where the coupling strength between the two resonators is usually larger than losses, the current responses in the two resonators exhibit two peaks above and below the resonant frequency of individual resonators, namely the even and odd mode, respectively. At the even mode, the currents in the two resonators are in phase, whilst $180^{\circ }$ out of phase at the odd mode^34^. Due to high Q-factor of the resonators in free space, the internal resistance of the resonators is negligible. By solving the natural response of the system, the splitting frequency can be approximated as (see Supplementary Information Note 2B).

2 $$\begin{aligned} f_{e,o} \approx \dfrac{\tilde{f_0}}{\mathfrak {R}\left[ \sqrt{1 \pm \dfrac{{\mathcal {M}}(\tilde{f_0})}{L_0 + \Delta {\mathcal {L}}'(\tilde{f_0})} -j \dfrac{ \Delta {\mathcal {L}}''(\tilde{f_0})}{L_0 + \Delta {\mathcal {L}}'(\tilde{f_0})} } \,\, \right] } \end{aligned}$$

where $f_{e,o}$ denotes the even and odd mode respectively, ${\mathcal {M}}(\tilde{f_0})$ and ${\mathcal {L}}(\tilde{f_0})$ are the complex coefficients at $\tilde{f_0}$, and $\tilde{f_0}$ is the resonant frequency of the resonator in the non-ideal medium. The resonant frequency inside the medium is the root of the following equation representing the total reactance equalling to zero: $\tilde{f_0}^2 \left( L_0 + \Delta {\mathcal {L}}'(\tilde{f_0}) \right) - 1/(4 \pi ^2 C_0) =0$. Hence, we compare the theoretical predictions of the splitting resonances to the simulation results in Fig. 3a. The simulation data is obtained by computing the current responses in both resonators from the circuit model in (1), given that the excitation voltage of 1 V is applied to one resonator. The odd and even mode are detected by finding the peaks in the current response of either coil. When the medium conductivity is relatively small ($\sigma _m<1$ S/m), the splitting frequencies are practically equal to those in the free space case. As the conductivity increases, the upper and lower resonances rise in the same fashion as that of a single isolated resonator in the medium. The mutual coupling tends to zero when the medium is very conductive, resulting in the merger of the two modes. In the simulations, at $\sigma _m \approx 4.9$ S/m, the current response exhibits only a peak at the higher resonance instead. The phenomenon is indeed due to the power absorption in the medium, resulting in a very low Q-factor of the system. The resonances may be still separated but the even mode is overshadowed by the slope of the odd mode as the eigenvalue method does not take into account the stretch of the current response of the resonators.

Next, we examine the current distribution in the two resonators versus frequency and medium conductivity, as shown in Fig. 3b,c. In contrast to the free space case, the almost perfect symmetry in the moduli of current responses in both resonators is remarkably broken in a surrounding non-ideal environment. Additionally, the decaying amplitude of the even mode with the increasing medium conductivity is higher compared to the odd mode. When the attenuation of the magnetic coupling is large enough, the eddy currents can completely suppress the lower resonant frequency. As it happens, each current response in the two coils displays an under-coupled-like region with only one peak. This phenomenon can also be observed in the phase difference of the current responses. The phase difference becomes more gradual since medium losses are added, which is often seen in coupled-resonator systems in free space with a low Q-factor. Differently from those systems, where the phase difference varies gently between 0 and $-\pi$ rad, the phase difference may unexpectedly move outside of that range with increasing conductivity, see Fig. 3c. As the transmission medium becomes strongly conductive, the phase difference tends to $-\pi$ corresponding to the sole odd mode in the current magnitude. Finally, the antisymmetry of the current responses indicates that resonant peaks undergo very different amounts of damping over the frequency range. The main reason for those phenomena is the additional phase to the circuit added via the complex mutual inductance, which can be shown with the circuit theory (see Supplementary Information Note 2C).

### Dispersion equation of MI waves

**Figure 4:**
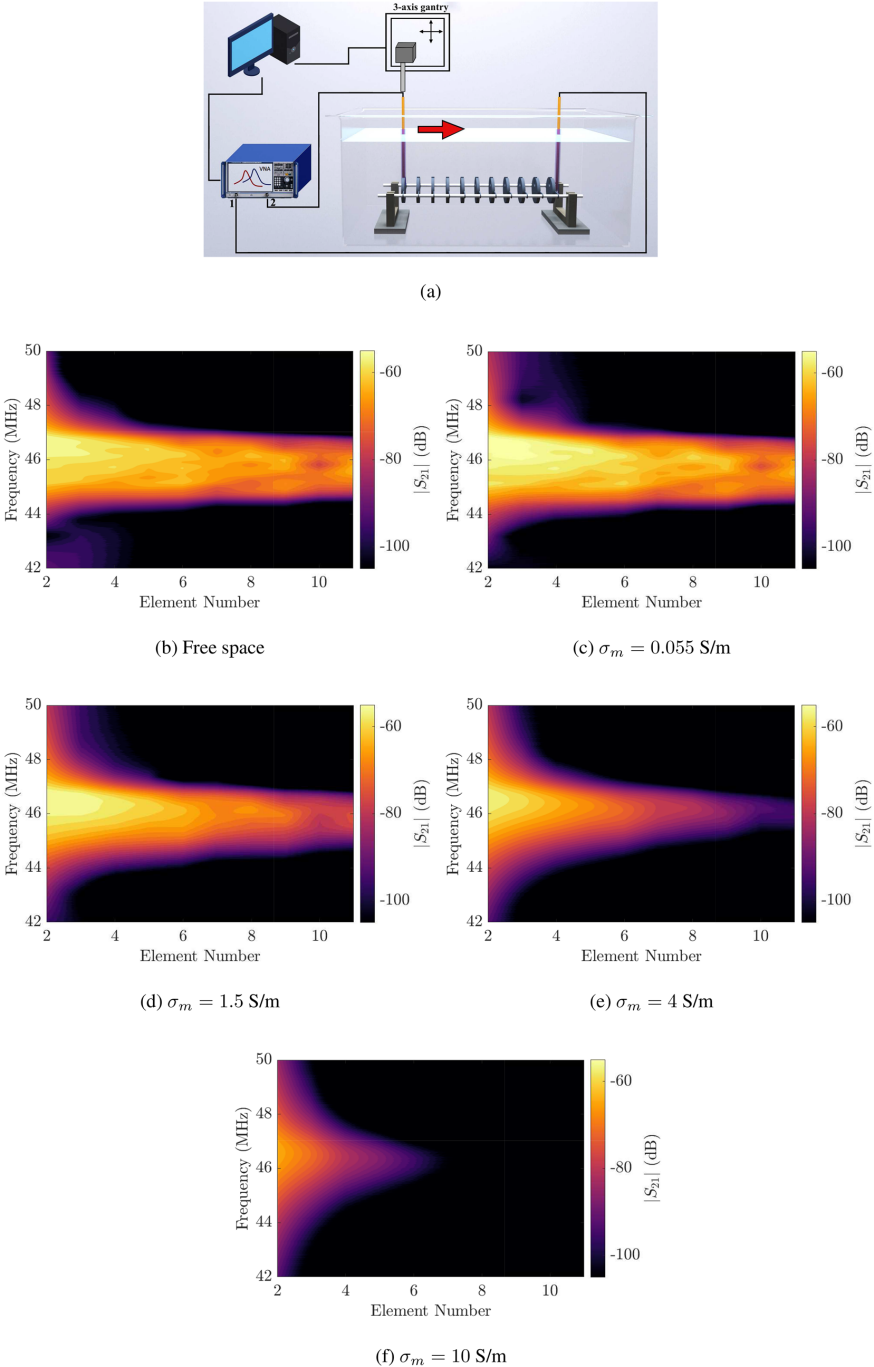
Laboratory experiments. (**a**) Schematic of the experimental apparatus. (**b**–**f**) Transmission coefficients, $S_{21}$, in dB scale measured along a coaxial 1-D waveguide as a function of the element position. The waveguide has an inter-element distance of $d=30$ mm, submerged in (**b**) free space and (**c**–**f**) an aqueous solution. The medium conductivity is (**b**) $\sigma _m=0.055$, (c) $\sigma _m=1.5$, (**d**) $\sigma _m=4$, and (**e**) $\sigma _m=10$ S/m.

Figure 4a illustrates an 1-D coaxial MI waveguide completely immersed in an infinite, linear, isotropic, homogeneous, and dissipative medium. Each element is a capacitively-loaded split ring resonator insulated by a small dielectric cavity to prevent current leakage to the environment. The dielectric cavities having little-to-no impact on the magnetic channels are assumed to be vacuum. The excitation is an external sinusoidal voltage $V_0$ applied to the first element of the MI waveguide. We assumed a time variation of the form $\exp (j\omega t)$ for all variables. To simplify the configuration, all elements in the chain are identical ($R_n = R$, $L_n = L$ and $C_n = C$). Consequently, a simple wave solution can be applied due to the attenuation of magnetic field, resulting current of the *n*-th coil in the form: $I_n=I_0e^{-n\gamma d}$ where $I_0$ is a constant, $\gamma =\alpha +j\beta$ is the propagation constant, $\alpha$ is the attenuation coefficient and $\beta$ is the phase constant, and *d* is the distance between two adjacent elements^8,9^. Finally, we derive the dispersion relationship from the wave solution above and Kirchhoff’s voltage law:

3 $$\begin{aligned} \frac{\omega _0^2}{\omega ^2}+\frac{j}{Q}\frac{\omega _0}{\omega }-\left( \frac{\Delta {\mathcal {L}}}{L} +1\right) = \sum _{n=1}^{p}\kappa _n \cosh {\left( n\gamma d\right) } \end{aligned}$$

where $\omega _0=1/\sqrt{LC}$ and $Q=\omega _0 L /R$ are the fundamental resonant frequency and the Q-factor of the capacitively-loaded split rings in free space, $\kappa _n=2 {\mathcal {M}}_n/L$ is the mutual coupling coefficient, ${\mathcal {M}}_n$ are the lossy mutual inductance between two elements located at a distance of *nd* apart from each other respectively, *p* is the upper limits of the summation, depending on how far the elements can interact magnetically before coupling becomes negligible. When the medium is vacuum, the lossy self-inductance and the added mutual inductance no longer exist, i.e., $\Delta {\mathcal {L}} = 0$ and ${\mathcal {M}} = M_0$ where $M_0$ are the mutual inductance in vacuum. Consequently, (3) reduces to the dispersion equation for MI waves in free-space^8,9^.

**Figure 5:**
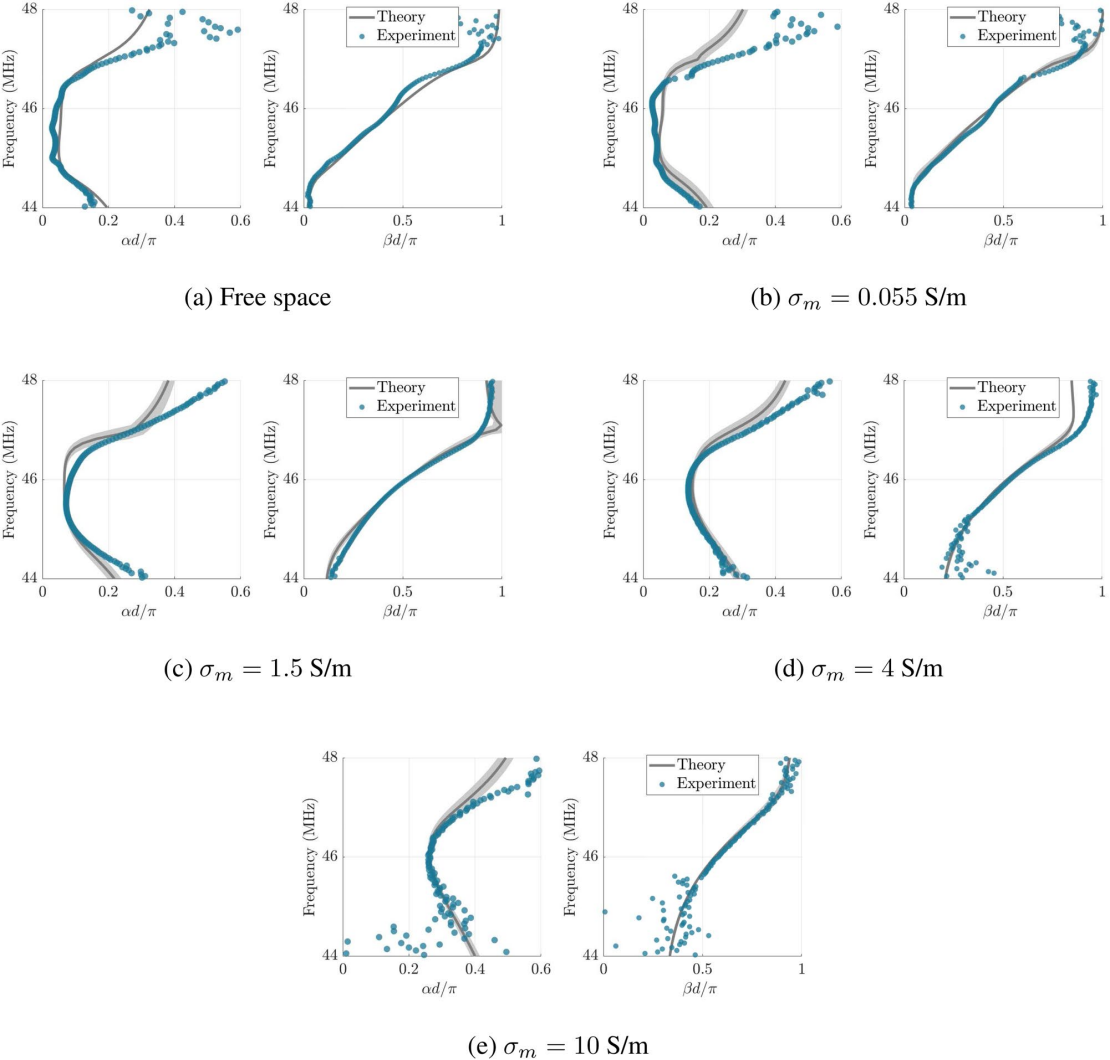
Dispersion relationship of MI waves in non-ideal media. Experimentally measured (dots) and theoretically calculated (solid curve) attenuation (left) and phase constant (right) of MI waves propagating in (**a**) free space and (**b**–**e**) an aqueous solution. The medium conductivity is (**b**) $\sigma _m=0.055$, (**c**) $\sigma _m=1.5$, (**d**) $\sigma _m=4$, and (**e**) $\sigma _m=10$ S/m. The shaded area represents the spread in the theoretical results due to the $7\%$ uncertainty attributed to the lossy Kirchhoff coefficients (${\mathcal {L}}$ and ${\mathcal {M}}$).

### Laboratory experiment

We made a waveguide sample consisting of 11 capacitively-loaded split rings of the same size as above with an inter-element distance of $d=30$ mm (Fig. 4a). Each split ring is insulated by a 3D-printed dielectric cavity, see Methods and Supplementary Information Note 3A. The split ring resonators with their corresponding cavities exhibit an average resonant frequency of $f_0=45.9$ MHz and Q-factor of $Q=117.4$. We measured the transmission coefficient, $S_{21}$, of the waveguide under test by using two magnetic probes connected to a Vector Network Analyser (VNA) (see Methods). Figure 4c–f illustrate the modulus of the measured transmission coefficient from 40 MHz ($\approx 0.9f_0$) to 52 MHz ($\approx 1.1f_0$) in logarithmic scale along the waveguide embedded in four different aqueous solutions. The solutions have the conductivity of $\sigma _m = 0.055$, 1.5, 4, 10 S/m similar to that of tap water, blood, seawater, massive sulfide minerals respectively^23–25,33^. We excluded the result for the first element of the waveguide due to the strong direct coupling between the transmitting and receiving probes. Unlike in free space (Fig. 4b), MI waves while penetrating in an attenuating medium suffer from larger attenuation. When the solution is weakly conductive ($\sigma _m = 0.055$ S/m, Fig. 4c), the MI waves can travel through the waveguide embedded in the dissipative medium within a bandwidth similar to that in free space. The bandwidth reduces rapidly with increasing conductivity which corresponds to stronger attenuating effects of the eddy currents. When the medium conductivity is $\sigma _m = 4$ S/m (Fig. 4e), the signals barely reach the tenth resonators (see Fig. S11 in Supplementary Information). To a greater degree, MI waves completely vanish after penetrating through 6 elements in the solution with conductivity of $\sigma _m = 10$ S/m (Fig. 4f). In addition, the centre of the passband comparatively moves up in the frequency spectrum due to the shift of the resonance of the split rings inside a conductive medium as discussed above.

By using the post-processing procedure (see Methods), the dispersion constants can be extracted from the transfer coefficients at elements about the middle of the waveguide. Figure 5 shows experimental results for the attenuation $\alpha d$ and phase constant $\beta d$ per unit cell for four different medium conductivities. They present the experimental data along with the theoretical values given by (3), considering up to the fifth-nearest-neighbour coupling. The analytical solutions for the lossy Kirchhoff coefficients yield an average relative difference of 7% compared to the full-wave simulation when the radius of the insulators is three times larger than the coil radius. The immersion level and the notch on the dielectric insulators are shown to only modify the those coefficients by a maximum relative deviation of 3% with respect to the simulation results for a infinite medium background and perfectly cylindrical insulators (see Fig. S6 in Supplementary Information). Thus, we encapsulate those uncertainties by attributing a 7% variability to both complex coefficients ($\Delta {\mathcal {L}}$ and ${\mathcal {M}}$).

Within the pass band, we find the experimental data and theoretical values for the phase constant in excellent agreement, although towards the edges of the passband there is some deviation. Meanwhile, the agreement for the attenuation diagram is satisfactorily good. We believe the discrepancy in the passband is due to the multiple fields on the measuring probe, and most importantly the strong effect of the eddy currents on the mutual coupling between the measuring probes and the split rings.

At the lower end of the passband, MI waves in an attenuating environment exhibit a non-zero phase constant due to the extra phase introduced by the eddy currents. It was reported in the literature that the mutual inductance in free space becomes complex in the presence of the retardation effect when the size of the structure is no longer electromagnetically small^35,36^. Physically, this complex mutual coupling results in power dissipation of MI waves. In the case of retardation effect in free space, it is radiation loss, while it is eddy current loss in our case. As can be seen in Fig. 5, the MI waves change from forward waves to backward waves at the upper edge of the passband. Previously, MI waves were reported to change their type from backward waves to forward waves in diatomic metamaterial structures with co-directional power flow, where one planar line is shifted by a half of the period of the other^37^. When the magnitude of the coupling constant between unit cells of two lines is larger than that within a unit cell of a line, forward MI waves occur for a certain range of frequency because the in-phase electromotive forces (emfs) can cancel out the out-of-phase ones. In our case, the existence of backward MI waves is entirely due to the phase of the complex mutual coupling (corresponding to the net phase of the emfs relative to their driving currents) rather than the disparity in modulus of the mutual couplings. The phase difference between currents in two neighbours is now not equal to $\beta d = \pi$ at the upper edge of the passband as in the free space case. The existence of backward MI waves is consistent with the phase difference between two coupled resonators shifting out of the range 0 and $-\pi$, illustrated in Fig. 3c, when the medium conductivity is non-zero. We note that if the phase difference between adjacent elements of the MI waveguide is nearer to $\pi$ than 0, backward MI waves exist, else forward MI waves. However, we cannot observe this interesting phenomenon when the MI waves switches from forward waves to backward waves in the measurements due to the noise becoming too significant near the edge of the passband.

Generally, excited eddy currents in the transmission medium alter the dispersion of MI waves. This feature may open up an opportunity to tailor the dispersion of MI waves by adjusting the phase of the coupling constants, which can be done by inserting non-ideal media with different permittivity, permeability and conductivity between elements of the waveguide. We expect power losses due to the medium is the trade-off in this scenario. In addition, by validating the general dispersion equation, we also obliquely corroborate the equivalent circuit model for two coupled coils in non-ideal media with the existence of the lossy Kirchhoff coefficients.

## Discussions

In a broader context, we have formulated an equivalent circuit model for two coupled resonators inside a homogeneous attenuating medium with the emergence of the complex Kirchhoff coefficients, arising from the impacts of eddy currents. By employing this circuit model, we have derived a general dispersion equation for MI waves in those environments. We experimentally demonstrated this equation for a 1-D axial waveguide made of capacitively-loaded split rings in an aqueous solution with four different conductivities. Our simulation results show that with increasing medium conductivity, MI waves can change from forward to backward waves at the higher branch of the stop band because of the extra phase shift the eddy currents introduce. Even though the turning point, where the MI waves switch their type, cannot be observed in the experiments due to severe signal-to-noise ratios in the branch of evanescent waves, our study may give a new degree of freedom to tailor the dispersion of MI waves based on the phase rather than the strength of the magnetic coupling, leading to unusual MI wave propagation behaviours. It can be engineered by inserting non-ideal media between waveguide elements. The additional losses introduced by the media may be the trade-off in this scenario.

Although we only determine the dispersion constants of an axial waveguide, the general dispersion is equally valid for planar waveguide configurations, which are immediately relevant to body area networks^38,39^, given that their complex Kirchhoff coefficients are known. We expect that the MI waves in planar waveguides may change their type at the edge of the passband as well, but from backward to forward waves, however, it is notable that the mathematical method to estimate the complex terms in this paper is indeed not applicable to the planar cases. When replacing the dielectric cavities by infinitely wide layers, the boundary value problem therefore becomes: an infinite broad layer of dielectric containing the resonators embedded in an infinite volume of a conductive dissipative environment. It would cause a significant inaccuracy in the prediction of complex coefficients.

Finally, we believe that our model can offer insights to the network analysis, design and optimization for any system employing magnetic induction or MI waves in arbitrary media, such as underground/underwater sensor networks, wearable/implanted sensors for biological systems, and wireless power transfer in harsh environments.

## Methods

### Simulation

Fully vectorial numerical simulations, from which all lossy Kirchhoff coefficients in (1) are extracted, are conducted using CST EM Studio (CST EMS). To simulate the change in the impedance of the transmitter as well as the change in the induced electromotive force at the receiver, the near-field feedback effects from the receiver must be neutralized. For this purpose, in the simulations, the transmitting coil is excited by using a lumped port injecting an alternating current of a constant amplitude of 1 A while the receiving coil is connected to a current port of 0 A. These ideal ports aim to eliminate the conduction current in the receiver, leaving only the induced electromotive force in the receiver to be measured, resulting in no feedback effect from the receiver to the transmitter.

4 $$\begin{aligned} \begin{bmatrix} V_1 \\ V_2 \end{bmatrix} = \begin{bmatrix} Z_0+j\omega {\mathcal {L}} &{} j\omega {\mathcal {M}} \\ j\omega {\mathcal {M}} &{} Z_0+j\omega {\mathcal {L}} \end{bmatrix} \begin{bmatrix} 1 \\ 0 \end{bmatrix} = \begin{bmatrix} Z_0+j\omega {\mathcal {L}} \\ j\omega {\mathcal {M}} \end{bmatrix} \end{aligned}$$

Then, we can extract the lossy Kirchhoff coefficients from the simulated voltages at the current ports by using (4). Please note that to accurately calculate the complex self inductance, solver order in CST EMS is set to 3rd (high accuracy) while adaptive mesh refinement is at least 10 passes.

### Sample fabrication

Since there is no natural solid material which has variable electrical conductivity suitable for measurements, the media in the experiments are made of aqueous solutions of sodium chloride (salt) with variable concentration. The conductivity is assessed with a conductivity-meter calibrated with standard calibration solutions (HANNA HI-70030 12880 $\mu$S/cm). The relative permittivity of the sodium chloride solutions follows that of deionized water, $\epsilon _r=78$, at room temperature at radio frequency^40,41^.

The tank containing the medium is made of polymethyl methacrylate with a dimension of 230 mm$\times 480$ mm$\times 260$mm. The transmitting and receiving probe is manufactured from a semirigid RG402/U 50 $\Omega$ coaxial cable with one end being a 10-mm-diameter loop and a 15-mm-diameter loop made from their inner conductors, respectively. The split rings were fabricated by slicing a cylindrical copper tube (diameter of 22 mm). Their dimensions are: mean radius of $r_0=11$ mm, width of $w=1$ mm, height of $l=5$ mm, and the gap width is $g=1$ mm, see Fig. 1b. The rings are then tuned to work at the resonant frequency of $f_0=46$ MHz by soldering nominally identical 330 pF capacitors of type of polystyrene film at the gap.

In order to obtain a reasonable match to the analytical conditions, the radius of the dielectric cavities is chosen to be three times the radius of the coil. To measure the near fields, enclosures were made with a small notch allowing probes to more closely approach the rings. We also attach two symmetric rings to the sides of the cavities for minimizing misalignment errors, as shown in Fig. S5a in the Supplementary Information. There is a small centrally-aligned hollow cylindrical pipe for fixing the position of split ring resonators. The dimensions of the dielectric cavities are as follows: a radius of $R = 33$ mm, height of $h = 7$ mm, and the diameter and the length of the notch are $D_n = 14$ mm and $l_n = 12$ mm, respectively. We realized the body and the cap of dielectric cavities by using 3D printing technique with acrylonitrile butadiene styrene (ABS) filament, see Fig. S7 in the Supplementary Information. To prevent water leakage, the dielectric cavities are sealed with silicone adhesive sealant (LOCTITE SI 595).

We characterised the fabricated resonating elements of the waveguide by two important parameters, the resonant frequency and Q-factor. The resonant frequency and the Q-factor of each split ring resonator with and without its insulating cavity are measured by using the transmission-type method in combination with Lorentzian fit (see Supplementary Information). Only the rings that exhibit a resonant frequency of $f_0=46$ MHz $\pm 0.5\%$ are used to construct MI waveguides.

### Measurement procedure

In order to minimize misalignment errors, we designed two symmetric rings attached to the sides of insulating cavities. The insulators were then interconnected via two polytetrafluoroethylene (PTFE) rods through these rings. We carefully aligned our MI waveguides within a $\pm 0.1$ mm accuracy along the rods. The whole frame was then mounted on U-shaped cantilevers that are fixed to the bed of the tank, as shown in Fig. 4a. The tank was then filled with sodium chloride solution with four different conductivities. In the experiments, we employed two magnetic loop probes connecting to two ports of a VNA (Keysight E5061B) to provide a transmitting-receiving scheme. The transmitting and receiving probes are manufactured from a semirigid RG402/U 50 $\Omega$ coaxial cable with one end being a 10-mm-diameter loop and a 15-mm-diameter loop made from their inner conductors, respectively. They are insulated using waterproof plastic covers to prevent direct contact with the solutions to improve the measurement accuracy. Throughout the experiments, the transmitting probe was placed centrally aligned behind the first coil to excite the MI waveguide whilst the receiving probe was attached to the arm of a computer-controlled 3-axis-gantry. Then, by using the gantry control software, we moved the receiving probe to scan the magnetic field near each coil, see Fig. 4a. In the measurement, the VNA recorded the scattering matrix between two magnetic probes for 1601 frequency points in the range from 40 MHz ($\approx 0.9f_0$) to 52 MHz ($\approx 1.1f_0$) with an averaging factor of 64.

### Post processing

The retrieving procedure to extract the propagation constants is as follows. We assumed that the transfer coefficient recorded by the measuring probes at the split ring *n* is proportional only to the current flowing in that ring, i.e., $S_{21|n} \propto I_n$. We also assumed that the MI waveguide supports two complex modes with two complex values of the propagation constant $\gamma d$. Considering two forward and two reflected waves at the end of the waveguide due to the mismatch condition, the measured transfer function for a probe located at the *n*-th element of the array can be rewritten in the form:

5 $$\begin{aligned} S_{21|n} \propto I_n=\left( a_1 e^{-n\gamma _1 d}+b_1 e^{n\gamma _1 d} \right) + \left( a_2 e^{-n\gamma _2 d}+b_1 e^{n\gamma _2 d} \right) \end{aligned}$$

where $a_1$, $a_2$, $b_1$, and $b_2$ are constant, and *d* is the distance between 2 elements. After a few algebraic operations, the value of $\gamma _1 d$ and $\gamma _2 d$ can be determined by considering the ratios between $S_{21}$ at ring *n* and its six neighbour resonators.

6 $$\begin{aligned} \left\{ \begin{array}{l} \cosh (\gamma _1 d)= \frac{1}{2} \left( \dfrac{2uv-u-w}{2 (2u^2-v-1)} \pm \sqrt{\left[ \dfrac{2uv-u-w}{2 (2u^2-v-1)} \right] ^2-4 \dfrac{(v+1)^2-u(3u+w)}{2 (2u^2-v-1)} } \right) \\ \cosh (\gamma _2 d)= \frac{1}{2} \left( \dfrac{2uv-u-w}{2 (2u^2-v-1)} \mp \sqrt{\left[ \dfrac{2uv-u-w}{2 (2u^2-v-1)} \right] ^2-4 \dfrac{(v+1)^2-u(3u+w)}{2 (2u^2-v-1)} } \right) \end{array}\right. \end{aligned}$$

where $u=(S_{21|n+1}+S_{21|n-1})/(2S_{21|n})$, $v=(S_{21|n+2}+S_{21|n-2})/(2S_{21|n})$, and $w=(S_{21|n+3}+S_{21|n-3})/(2S_{21|n})$. One of two modes is the propagation mode while the other is non-propagating. It is worth noting that this method is best utilized for seven elements about the middle of the waveguides because the measurements at the beginning of the waveguide are strongly affected by the direct coupling between the transmitting and receiving probes while those towards the end of the line are strongly affected by reflected waves from the unmatched condition.

## Supplementary Information

Supplementary Information
