# Supplementary material for: Magnetoinductive waves in attenuating media
Source: Sci Rep. 2021 Apr 7;11:7679. doi: 10.1038/s41598-021-85838-7 (PMC8027633; doi:10.1038/s41598-021-85838-7)
Supplement: Supplementary file 1 — Supplementary Information [file 41598_2021_85838_MOESM1_ESM.pdf]

Magnetoinductive Waves in Attenuating Media Supplementary Information

Son Chu[1], Mark S. Luloff[2], Jiaruo Yan[1], Pavel Petrov[3], Christopher J. Stevens[1] & Ekaterina Shamonina[1]

[1]*Department of Engineering Science, University of Oxford, Oxford, OX1 3PJ, UK*

[2]*Canadian Nuclear Laboratories, Chalk River, ON K0J 1J0, Canada*

[3]*Department of Physics and Astronomy, University of Exeter, Exeter, EX4 4QL, UK*

1 Supplementary Note 1: Circuit Model for Two Coupled Coils Immersed inside a Homogeneous Attenuating Medium

1A. Complex Kirchhoff Coefficient Formulation We investigated the configuration, whose the cross-section is shown in Fig. S1a: two coils with the same orientation separated by a distance from each other are embedded in a homogeneous dissipative medium. The coils are insulated from the surrounding medium by placing them inside small dielectric cavities to prevent current leakage. To simplify the configuration, these cavities made of non-magnetic insulating material have little-to-none effect on the magnetic channels and thus are assumed to be free space. To calculate the complex coefficients, the most general approach is to formulate the differential equation for the magnetic vector potential, which describes the electromagnetic properties of the system. The dielectric cavities are then replaced by infinitely wide dielectric layers to derive a closed-form analytical solution for the magnetic vector potential. Thus, boundary value problem is divided into

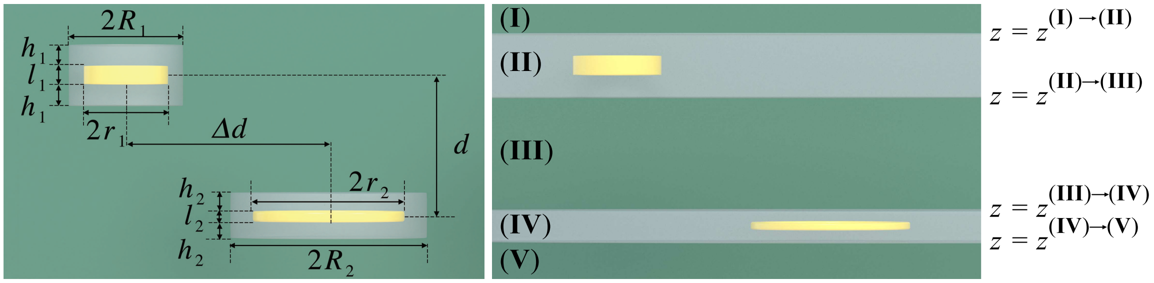

(a) (b)

Figure S1: The cross section of the configuration investigated consists of two circular coils spaced apart a certain distance in a conductive medium. (a) The finite size dielectric enclosures used in the actual configuration for coil insulation, in the theoretical model (b) are considered as infinite dielectric layers.

the five regions labelled with Roman numerals, as shown in Fig. S1b.

Notation: Recall that the subscripts 1 and 2 denote coil 1 and 2, the superscript denote the layers in Fig. S1b, r, l, w and N are the mean radius, thickness, width and number of turns of the coil respectively, as shown in Fig. S1a, d and Δd are the vertical distance and horizontal displacement between two coils, h is the separation from the surface of a coil to the surface of its respective insulating box, $c = d - (h_1 + h_2) - (t_1 + t_2)/2$ is the thickness of conductive layer between two coils, J_i is the Bessel function of the first kind, of the i-th order, t is the time variable.

Here, the superposition principle is applied to reduce the complexity. The field solutions for a coil are formulated in the absence of the other. Similar to most eddy current problems, cylindrical coordinates are employed in this case to facilitate the boundary conditions. All the media are

assumed to be linear, isotropic and homogeneous. In this case, we derive the differential equation of the magnetic vector potential $\mathbf{A}^{(n)}$ as[1]:

$$\nabla^2 \mathbf{A}^{(n)} = \begin{bmatrix} \partial_z^2 A_\rho^{(n)} + \partial_\rho^2 A_\rho^{(n)} + \frac{1}{\rho}\partial_\rho A_\rho^{(n)} - \frac{A_\rho^{(n)}}{\rho^2} + \frac{1}{\rho^2}\partial_\varphi^2 A_\rho^{(n)} - \frac{2}{\rho^2}\partial_\varphi A_\varphi^{(n)} \\ \partial_z^2 A_\varphi^{(n)} + \partial_\rho^2 A_\varphi^{(n)} + \frac{1}{\rho}\partial_\rho A_\varphi^{(n)} - \frac{A_\varphi^{(n)}}{\rho^2} + \frac{1}{\rho^2}\partial_\varphi^2 A_\varphi^{(n)} + \frac{2}{\rho^2}\partial_\varphi A_\rho^{(n)} \\ \partial_z^2 A_z^{(n)} + \partial_\rho^2 A_z^{(n)} + \frac{1}{\rho}\partial_\rho A_z^{(n)} + \frac{1}{\rho^2}\partial_\varphi^2 A_z^{(n)} \end{bmatrix}^{\boldsymbol{T}} \begin{bmatrix} \widehat{\boldsymbol{\rho}} \\ \widehat{\boldsymbol{\varphi}} \\ \widehat{\boldsymbol{z}} \end{bmatrix} \tag{S1}$$

$$= \mu^{(n)}\sigma^{(n)}\frac{\mathrm{d}\,\mathbf{A}^{(n)}}{\mathrm{d}\,t} + \mu^{(n)}\epsilon^{(n)}\frac{\mathrm{d}^2\,\mathbf{A}^{(n)}}{\mathrm{d}\,t^2}$$

where $[\mathbf{X}]^{\boldsymbol{T}}$ denotes the transpose matrix of a matrix $[\mathbf{X}]$, ρ, φ, and z are the radial, azimuthal, and axial coordinates, $\widehat{\boldsymbol{\rho}}$, $\widehat{\boldsymbol{\varphi}}$, and $\widehat{\boldsymbol{z}}$ are the corresponding unit vectors in cylindrical coordinates, μ_0 is the permeability of free space, $\mu^{(n)}$, $\epsilon^{(n)}$, and $\sigma^{(n)}$ are the permeability, permittivity, and conductivity of the n-th region respectively, and t is the time variable. Basically, the induced eddy current density must be in the same direction as the magnetic vector potential. With the assumption of an axially symmetric conductor geometry, the induced eddy currents have only one spatial component along the azimuthal angle φ and so is the magnetic vector potential, i.e., $\mathbf{A}^{(n)}(0, A_\varphi^{(n)}, 0)$ and $\partial\mathbf{A}^{(n)}/\partial\varphi = 0$. Thus, the differential equation for the magnetic vector potential of coil 1 due to an applied voltage V_0 reduces to:

$$\begin{aligned} \nabla^2 \mathbf{A}_1^{(n)} &= \left(\frac{\partial^2 A_{1\varphi}^{(n)}}{\partial z^2} + \frac{\partial^2 A_{1\varphi}^{(n)}}{\partial \rho^2} + \frac{1}{\rho}\frac{\partial A_{1\varphi}^{(n)}}{\partial \rho} - \frac{A_{1\varphi}^{(n)}}{\rho^2}\right)\widehat{\boldsymbol{\varphi}} \\ &= \left(\mu^{(n)}\sigma^{(n)}\frac{\mathrm{d}\,A_{1\varphi}^{(n)}}{\mathrm{d}\,t} + \mu^{(n)}\epsilon^{(n)}\frac{\mathrm{d}^2\,A_{1\varphi}^{(n)}}{\mathrm{d}\,t^2}\right)\widehat{\boldsymbol{\varphi}} \end{aligned} \tag{S2}$$

where $A_{1\varphi}^{(n)}$ is the vector potential of coil 1 in the region n, which for symmetry reasons only depends on the cylindrical coordinates ρ and z. To simplify the mathematical complexity, we define the magnetic vector potential in time-domain as the convolution of the Magnetic Vector

Potential Impulse Response (MVPIR) function with the transient current in the respective coils[2].

$$A_1(\rho,z,t) \equiv \widehat{A}_1(\rho,z,t) * i_1(t) \tag{S3}$$

where

$$\widehat{A}_1(\rho,z,t) = \widehat{E}_1(\rho,z,t) + \widehat{\Psi}_1(\rho,z,t) = \widehat{E}_1(\rho,z,t) + \widehat{\Psi}_1(\rho,z)\,\delta(t)$$

are the MVPIRs, $\widehat{\Psi}_1(\rho,z,t)$ is the MVPIR generated by a unit impulse current in coil 1 in free space, $\widehat{E}_1(\rho,z,t)$ is the MVPIR generated by eddy currents in the medium, and $\delta(x)$ is the Dirac delta function of variable x. The magnetic vector potential produced by a Dirac-coil (an infinitely thin coil with infinite conductivity) of radius r, is calculated in terms of an infinite integral of Bessel functions[3]:

$$\boldsymbol{\Psi_\delta}(\rho,z,t) = i(t)\frac{\mu}{2}\int_0^\infty r\,\mathrm{J}_1(\alpha r)\,\mathrm{J}_1(\alpha\rho)\,e^{-\alpha|z-z'|}\,\mathrm{d}\alpha \tag{S4}$$

By substituting $i(t)$ for $\delta(t)$ and taking the integral sum of all the Dirac-delta contributions over the volume of the coil 1 and then by multiplying the term by its appropriate turn density, the time-dependent unit impulse response of coil 1 can be determined as:

$$\widehat{\Psi}_1(\rho,z,t) = \frac{\mu^{(\mathbf{II})}\,\delta(t)\,N_1}{2\,w_1\,l_1}\int_0^\infty\int_{r_1-w_1/2}^{r_1+w_1/2}\int_{(-h_1-l_1)}^{(-h_1)} r\,\mathrm{J}_1\left(\alpha r\right)\mathrm{J}_1(\alpha\rho)\,e^{-\alpha|z-z'|}\,\mathrm{d}z'\,\mathrm{d}r\,\mathrm{d}\alpha \tag{S5}$$

Taking the Fourier Transform in t and Hankel Transform in ρ, the unit impulse response of coils can be rewritten in frequency domain and α-space:

$$\widehat{\Psi}_1(\alpha,z,\omega) = \frac{\mu^{(\mathbf{II})}\,N_1}{2\,\alpha\,w_1\,l_1}\int_{r_1-w_1/2}^{r_1+w_1/2}\int_{(-h_1-l_1)}^{(-h_1)} r\,\mathrm{J}_1\left(\alpha r\right)\,e^{-\alpha|z-z'|}\,\mathrm{d}z'\,\mathrm{d}r \tag{S6}$$

where ω and α are the transformed variables of t and ρ, respectively. Next, we derive the unit impulse responses induced by eddy currents in n-th region, $\widehat{E}_1^{(n)}(r, z, t)$, from the field solutions:

$$\left(\frac{\partial^2}{\partial z^2} + \frac{\partial^2}{\partial r^2} + \frac{1}{\rho}\frac{\partial}{\partial \rho} - \frac{1}{\rho^2}\right) \widehat{E}_1^{(n)}(\rho, z, t) = \left(\mu^{(n)}\sigma^{(n)}\frac{\mathrm{d}}{\mathrm{d}\,t} + \mu^{(n)}\epsilon^{(n)}\frac{\mathrm{d}^2}{\mathrm{d}\,t^2}\right) \widehat{E}_1^{(n)}(\rho, z, t) \quad \text{(S7)}$$

Applying the Fourier Transform and Hankel Transform,

$$\frac{\partial^2}{\partial z^2}\widehat{E}_1^{(n)}(\alpha, z, \omega) = \left(\alpha^2 + j\omega\mu^{(n)}\sigma^{(n)} - \omega^2\mu^{(n)}\epsilon^{(n)}\right) \widehat{E}_1^{(n)}(\alpha, z, \omega) \quad \text{(S8)}$$

The solutions of equation (S8) can be expressed in the general form:

$$\widehat{E}_1^{(n)}(\alpha, z, \omega) = X_1^{(n)}(\alpha, \omega)\, e^{\alpha_n z} + Y_1^{(n)}(\alpha, \omega)\, e^{-\alpha_n z} \quad \text{(S9)}$$

where $\alpha^{(n)} = \sqrt{\alpha^2 + j\omega\mu^{(n)}\sigma^{(n)} - \omega^2\mu^{(n)}\epsilon^{(n)}}$ is the separation constant in the n-th zone, and $X_{1,2}^{(n)}(\alpha, \omega)$ and $Y_{1,2}^{(n)}(\alpha, \omega)$ are unknown constants. In this particular configuration, cf. Fig. S1b, we have:

$$\begin{cases} \alpha^{(\mathbf{II})} = \alpha^{(\mathbf{IV})} = \sqrt{\alpha^2 - \omega^2\mu^{(\mathbf{II})}\epsilon^{(\mathbf{II})}} \\ \alpha^{(\mathbf{I})} = \alpha^{(\mathbf{III})} = \alpha^{(\mathbf{V})} = \sqrt{\alpha^2 + j\omega\mu^{(\mathbf{I})}\sigma^{(\mathbf{I})} - \omega^2\mu^{(\mathbf{I})}\epsilon^{(\mathbf{I})}} = \sqrt{\alpha^2 + j\omega\mu_m\sigma_m - \omega^2\mu_m\epsilon_m} \end{cases} \quad \text{(S10)}$$

where μ_m, ϵ_m, and σ_m are the medium's relative permeability, relative permittivity and conductivity, respectively. Finally, the solutions for MVPIR for coil 1 problem in each region are summarized below:

$$\widehat{A}_1(\alpha, z, \omega) = \begin{cases} \widehat{\Psi}_1(\alpha, z, \omega) + X_1^{(\mathbf{II})}(\alpha, \omega)\, e^{\alpha^{(\mathbf{II})} z} + Y_1^{(\mathbf{II})}(\alpha, \omega)\, e^{-\alpha^{(\mathbf{II})} z}, & \text{for n} = \mathbf{II}, \\ X_1^{(n)}(\alpha, \omega)\, e^{\alpha^{(n)} z} + Y_1^{(n)}(\alpha, \omega)\, e^{-\alpha^{(n)} z}, & \text{for n} \neq \mathbf{II}. \end{cases} \quad \text{(S11)}$$

We then determine the unknown coefficients in (S11) by applying the boundary conditions (S12) and (S13) from the continuity conditions for the electric and magnetic field components at the

interfaces shown in (S14)

$$\widehat{A}^{(n)}(\alpha, z, \omega)\Big|_{z=z^{n\to n+1}} = \widehat{A}^{(n+1)}(\alpha, z, \omega)\Big|_{z=z^{n\to n+1}} \tag{S12}$$

$$\frac{1}{\mu^{(n)}}\frac{\mathrm{d}\,\widehat{A}^{(n)}(\alpha, z, \omega)}{\mathrm{d}\,z}\Bigg|_{z=z^{n\to n+1}} = \frac{1}{\mu^{(n+1)}}\frac{\mathrm{d}\,\widehat{A}^{(n+1)}(\alpha, z, \omega)}{\mathrm{d}\,z}\Bigg|_{z=z^{n\to n+1}} \tag{S13}$$

$$z^{n\to n+1} = \begin{cases} -2h_1 - l_1, & \text{for interface } \textbf{I-II}, \\ 0, & \text{for interface } \textbf{II-III}, \\ c, & \text{for interface } \textbf{III-IV}, \\ c + 2h_2 + l_2, & \text{for interface } \textbf{IV-V}. \end{cases} \tag{S14}$$

where $z^{n\to n+1}$ is the location of the interface between consecutive zones n and $n+1$. Once all the coefficients are known, every other physically observable electromagnetic quantity can be calculated directly from the magnetic vector potential.

Here, we define the complex self inductance term in the same manner as the self inductance. Whereas the self inductance represents the interaction between the source magnetic field produced by a coil with itself to produce an EMF, the complex self inductance can be referred as the interaction of the secondary magnetic field generated by the eddy currents with the coil. On the other hand, since the magnetic vector potential in the region of the second coil is an aggregate of the primary field generated by the first coil and the secondary field generated by the eddy currents, cf. (S11), the complex mutual inductance is a summation of the original mutual inductance in free-space and the coupling between the second coil and the eddy currents generated by the first coil. In other words, it is the mutual inductance between two coils through the medium.

Consequently, we calculate the complex self inductance of coil 1 by integrating the eddy-current-based MVPIRs arising from currents in coil 1 in the region containing the coil itself over its volume.

$$\Delta\mathcal{L}_1 = \frac{2\pi N_1}{w_1\, l_1} \int\limits_0^\infty \int\limits_{r_1-w_1/2}^{r_1+w_1/2} \int\limits_{(-h_1-l_1)}^{(-h_1)} \alpha\, r\, \mathrm{J}_1\left(\alpha r\right)\, \widehat{E}_1^{(\mathbf{II})}(\alpha, z, \omega)\, \mathrm{d}z\, \mathrm{d}r\, \mathrm{d}\alpha \tag{S15}$$

Likewise, we obtain the complex mutual inductance by integrating the eddy-current-based MVPIR arising from currents in a nearby coil in the region containing the secondary coil over its volume. Applying integral transformations in [3,4] for the mutual inductance of two coils with a horizontal offset, the complex mutual inductance can be calculated by:

$$\mathcal{M} = \mathcal{M}_{21} = \frac{2\pi N_2}{w_2\, l_2} \int\limits_0^\infty \int\limits_{r_2-w_2/2}^{r_2+w_2/2} \int\limits_{c+h_2}^{c+h_2+l_2} \alpha\, r\, \mathrm{J}_0\left(\alpha \Delta d\right)\, \mathrm{J}_1\left(\alpha r\right)\, \widehat{E}_1^{(\mathbf{IV})}(\alpha, z, \omega)\, \mathrm{d}z\, \mathrm{d}r\, \mathrm{d}\alpha \tag{S16}$$

By proceeding analogously, the solutions for MVPIR for coil 2 problem in each region are obtained:

$$\widehat{A}_2(\alpha, z, \omega) = \begin{cases} \widehat{\Psi}_2(\alpha, z, \omega) + X_2^{(\mathbf{IV})}(\alpha, \omega)\, e^{\alpha^{(\mathbf{II})} z} + Y_2^{(\mathbf{IV})}(\alpha, \omega)\, e^{-\alpha^{(\mathbf{II})} z}, & \text{for n} = \mathbf{IV}, \\ X_2^{(n)}(\alpha, \omega)\, e^{\alpha^{(n)} z} + Y_2^{(n)}(\alpha, \omega)\, e^{-\alpha^{(n)} z}, & \text{for n} \neq \mathbf{IV}. \end{cases} \tag{S17}$$

where

$$\widehat{\Psi}_2(\alpha, z, \omega) = \frac{\mu^{(\mathbf{II})}\, N_2}{2\, \alpha\, w_2\, l_2} \int\limits_{r_2-w_2/2}^{r_2+w_2/2} \int\limits_{c+h_2}^{c+h_2+l_2} r\, \mathrm{J}_1\left(\alpha r\right)\, e^{-\alpha|z-z'|}\, \mathrm{d}z'\, \mathrm{d}r, \tag{S18}$$

and $X_2^{(n)}(\alpha, \omega)$ and $Y_2^{(n)}(\alpha, \omega)$ can be found by solving the boundary conditions (S12) – (S14). Finally, we can obtain the complex self inductance of coil 2 and the complex mutual inductance between coil 2 and coil 1 by using the following equations:

$$\Delta\mathcal{L}_2 = \frac{2\pi N_2}{w_2\, l_2} \int\limits_0^\infty \int\limits_{r_2-w_2/2}^{r_2+w_2/2} \int\limits_{c+h_2}^{c+h_2+l_2} \alpha\, r\, \mathrm{J}_1\left(\alpha r\right)\, \widehat{E}_2^{(\mathbf{IV})}(\alpha, z, \omega)\, \mathrm{d}z\, \mathrm{d}r\, \mathrm{d}\alpha \tag{S19}$$

$$\mathcal{M} = \mathcal{M}_{12} = \frac{2\pi N_1}{w_1\, l_1} \int\limits_{0}^{\infty} \int\limits_{r_1 - w_1/2}^{r_1 + w_1/2} \int\limits_{(-h_1 - l_1)}^{(-h_1)} \alpha\, r\, \mathrm{J}_0\left(\alpha \Delta d\right)\ \mathrm{J}_1\left(\alpha r\right)\ \widehat{E}_2^{(\mathbf{II})}(\alpha, z, \omega)\, \mathrm{d}z\, \mathrm{d}r\, \mathrm{d}\alpha \tag{S20}$$

1B. Simulation Results The two coils are identical split ring resonators with the dimension: mean radius $r_0 = 11$ mm, length $l = 5$ mm, width $w = 1$ mm, and gap width $g = 1$ mm. Their complex self inductance and mutual inductance can be recast as:

$$\Delta\mathcal{L} = \frac{2\pi N}{w\, l} \int\limits_{0}^{\infty} \int\limits_{r - w/2}^{r + w/2} \int\limits_{-h - l}^{-h} \alpha\, r\, \mathrm{J}_1\left(\alpha r\right)\ \widehat{E}_1^{(\mathbf{II})}(\alpha, z, \omega)\, \mathrm{d}z\, \mathrm{d}r\, \mathrm{d}\alpha \tag{S21}$$

$$\mathcal{M} = \frac{2\pi N}{w\, l} \int\limits_{0}^{\infty} \int\limits_{r - w/2}^{r + w/2} \int\limits_{c + h}^{c + h + l} \alpha\, r\, \mathrm{J}_0\left(\alpha \Delta d\right)\ \mathrm{J}_1\left(\alpha r\right)\ \widehat{E}_1^{(\mathbf{IV})}(\alpha, z, \omega)\, \mathrm{d}z\, \mathrm{d}r\, \mathrm{d}\alpha \tag{S22}$$

The medium now has relative permittivity of $\epsilon_r = 78$, relative permeability of $\mu_r = 1$, and variable conductivity. Firstly, we investigate the variation of the complex coefficients across a range of common medium conductivity and working frequency. The two coupled coils are then placed at a vertical distance of $d = 30$ mm and horizontal separation of $\Delta d = 0$ mm apart from each other inside a conductive medium. Fig. S2 compares the real and imaginary parts of the calculated complex coefficients obtained with the analytical solutions and the simulated results obtained with CST EMS. The complex Kirchhoff coefficients are a surface function of the working frequency and the medium conductivity. As is apparent from Fig. S2, the theoretical results calculated with (S21) and (S22) show the same trends as those determined with CST EMS. Both complex Kirchhoff coefficients are truly complex and frequency-dependent as confirmed by simulated data.

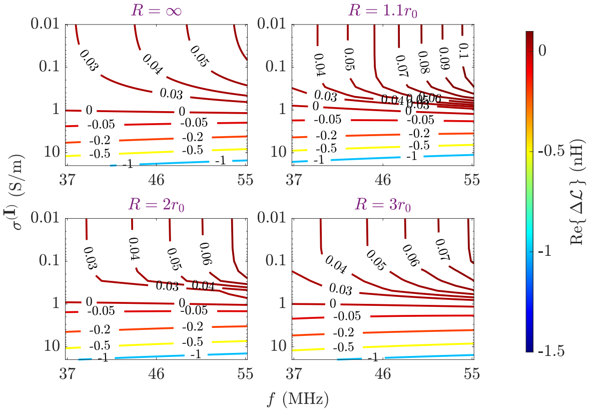

(a) The real part of $\Delta\mathcal{L}$

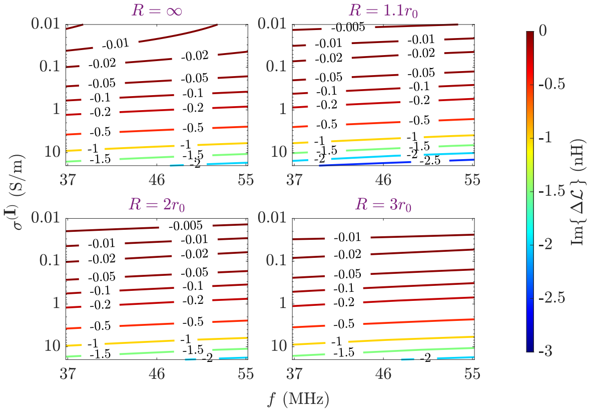

(b) The imaginary part of $\Delta\mathcal{L}$

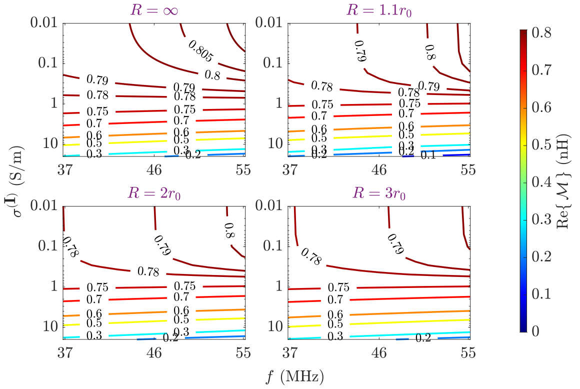

(c) The real part of $\mathcal{M}$

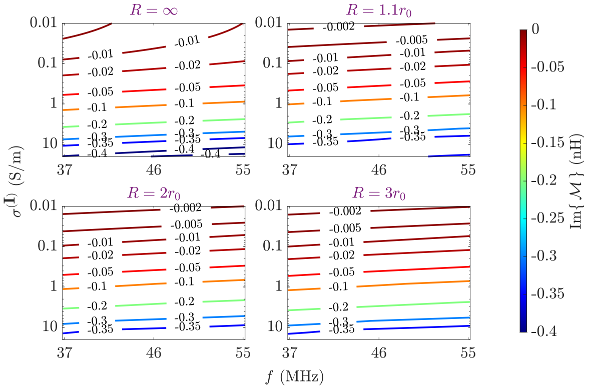

(d) The imaginary part of $\mathcal{M}$

Figure S2: Complex Kirchhoff coefficients $\Delta\mathcal{L}$ and $\mathcal{M}$ as a surface function of the working frequency and the medium conductivity. The medium conductivity is from $\sigma^{(\mathbf{I})} = 0.01$ to $\sigma^{(\mathbf{I})} = 20$ S/m while the working frequency ranges between $f = 37$ to $f = 55$ MHz. In each sub-figure, (Top left) the analytical values ($R = \infty$) are compared to CST EMS simulation results for (Top right) $R = 1.1r_0$, (Bottom left) $R = 2r_0$, and (Bottom right) $R = 3r_0$.

Both complex Kirchhoff coefficients exhibit an understandable frequency-dependent feature. For simplicity and without loss of generality, all results presented next are calculated at the fre-

quency $f = 46$ MHz unless stated otherwise. Now the medium conductivity is set to $\sigma^{(\mathbf{I})} = 4$ S/m. Fig. S3 shows the complex Kirchhoff coefficients as a function of the normalised vertical distance between the two coils. Similar to Fig. S2, a set of three numerical simulations corresponding to three different dielectric cavity radii is examined: $R = 1.1r_0$, $2r_0$, and $3r_0$. Again, the analytical solutions (S21) and (S22) appear to constitute good approximations.

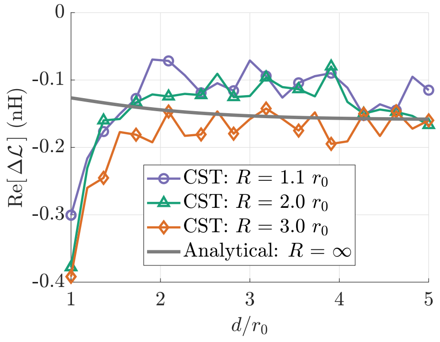

(a) The real part of $\Delta\mathcal{L}$

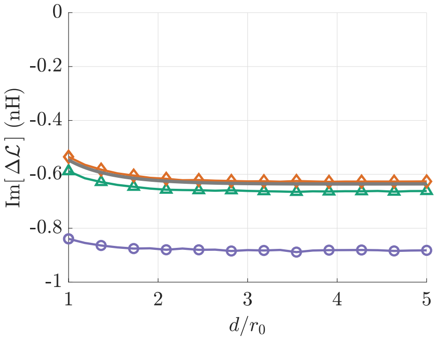

(b) The imaginary part of $\Delta\mathcal{L}$

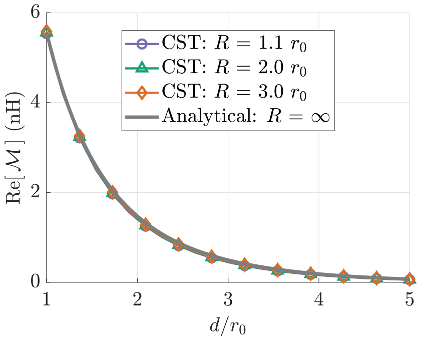

(c) The real part of $\mathcal{M}$

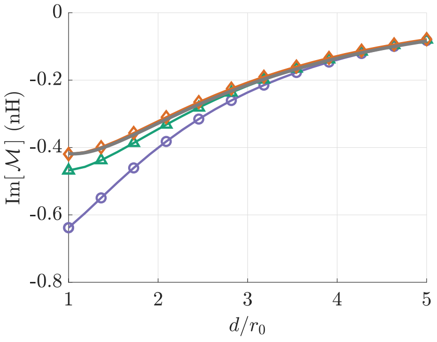

(d) The imaginary part of $\mathcal{M}$

Figure S3: Complex Kirhhoff coefficients $\Delta\mathcal{L}$ and $\mathcal{M}$ as a function of the normalised vertical separation d/r_0. Medium conductivity is $\sigma^{(\mathbf{I})} = 4$ S/m. The horizontal displacement between the two coils is $\Delta d = 0$ mm. CST EMS simulation results for three different sizes of the insulators: $R = 1.1r_0$, $2r_0$, and $3r_0$ are compared with those obtained with the analytical solutions.

However, when distance between two inductive circuits is small, i.e., less than $d < 1.5r_0$ for that particular coil arrangement, the analytical solutions show the validity for estimating correctly the complex mutual inductance and the imaginary part of the complex self inductance, but underestimate the magnitude of its real part. The main reason for this is that in the finite case, the portion of the conductive environment immediately adjacent to the dielectric cavities carries a significant amount of eddy currents near the two coils, which is not present in the infinite case. As a result, more losses are present as represented by the absolute value of the complex self inductance's real part when the distance of the two coils are close. It is a limitation of the analytical model where the infinite long layers of dielectric cavities are considered. Notably, the huge oscillations exhibiting in the real part of the complex self inductance are due to the computational imperfection in the finite-element simulations. Because of the relatively small electrical size of the coils in this configuration, the mesh in CST seems to be not dense enough to provide accurate approximation for the small real part of the complex self inductance, around a few hundred pH. These oscillations can be improved by further increasing the adaptive mesh refinement in CST EMS but the trade-off will surely be a very long computational time.

Finally, the validity of the analytical solutions for the complex Kirchhoff coefficients is examined in the situation that the receiving coil is displaced by a distance Δd away from the coaxial alignment. Fig. S4 presents the simulation results for the complex Kirchhoff coefficients as a function of the normalised horizontal displacement between the two coils. The analytical solutions succeed in providing a good approximation to the complex coefficients. Specially, (S21) is capable of correctly reproducing the interesting behaviour of the complex mutual inductance as a function

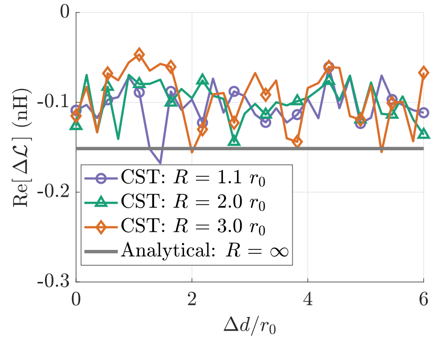

(a) The real part of $\Delta\mathcal{L}$

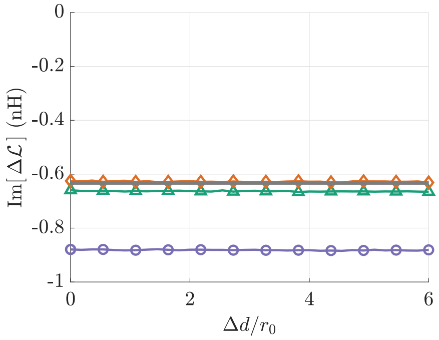

(b) The imaginary part of $\Delta\mathcal{L}$

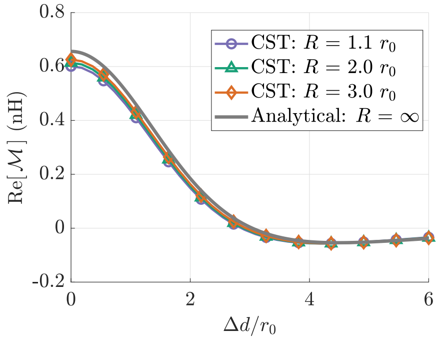

(c) The real part of $\mathcal{M}$

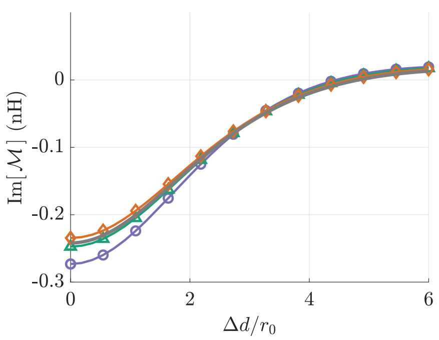

(d) The imaginary part of $\mathcal{M}$

Figure S4: Complex Kirhhoff coefficients $\Delta\mathcal{L}$ and $\mathcal{M}$ as a function of the normalised horizontal displacement $\Delta d/r_0$. Medium conductivity is $\sigma^{(\mathbf{I})} = 4$ S/m. The vertical distance between the two coils is $d = 30$ mm. CST EMS simulation results for three different sizes of the insulators: $R = 1.1r_0$, $2r_0$, and $3r_0$ are compared with those obtained with the analytical solutions.

of coil horizontal displacement, which exhibits oscillations in the magnitude with local minima and maxima without vanishing as in free space case. For the explanation, see [5,6].

2 Network Analysis

2A. Resonant Frequency and Q-factor When embedding a resonator circuit in a conductive medium, the induced eddy currents introduce additional losses and change the net self-flux of the circuit. As a result, we can observe a change in the resonant frequency, f_0 and a reduction in the quality factor, Q, of the circuit. Here, we use notations as follows: x is the circuit parameter of the coil in free space while $\tilde{x}$ is that of the coil inside a dissipative medium. The impedance of a coil when immersed in a medium background can be expressed by:

$$\tilde{Z} = Z_0 + j\omega\Delta\mathcal{L} = (R_0 + \omega\Delta\mathcal{L}'') + j\omega(L_0 + \Delta\mathcal{L}') + \frac{1}{j\omega C_0} \tag{S23}$$

where Z,R_0, L_0 and C_0 are the self impedance, resistance, inductance, capacitance of the coils in free-space, respectively, and $\Delta\mathcal{L} = \Delta\mathcal{L}' - \Delta\mathcal{L}''$ is the complex self inductance. In principle, the resonant frequency occurs when the inductive reactance cancels out the capacitive reactance. Unlike the normal lumped elements which are idealised to be frequency-independent for a certain range of frequency, the complex Kirchhoff coefficients are instead frequency-dependent. Therefore, the resonant frequency of a coil immersed inside a conductive medium is the root of the following equation representing the total reactance equalling to zero:

$$2\pi\tilde{f}_0\left(L_0 + \Delta\mathcal{L}'(\tilde{f}_0)\right) = \frac{1}{2\pi\tilde{f}_0 C_0} \Leftrightarrow \tilde{f}_0^{\,2}\left(L_0 + \Delta\mathcal{L}'(\tilde{f}_0)\right) - \frac{1}{4\pi^2 C_0} = 0 \tag{S24}$$

Hence, its Q-factor inside the medium, $\tilde{Q}$, is then obtained by:

$$\tilde{Q} = \frac{2\pi\tilde{f}_0\left(L_0 + \Delta\mathcal{L}'(\tilde{f}_0)\right)}{R_0 + 2\pi\tilde{f}_0\Delta\mathcal{L}''(\tilde{f}_0)} \tag{S25}$$

2B. Frequency Splitting The resonance splitting occurs when the two magnetically-coupled circuits are placed in the strongly-coupled regime, i.e., the strength of the mutual coupling between the two resonators is larger than losses [7]. The current responses in the two coils then exhibit two peaks above and below the resonance frequency in the non-coupled case, namely the odd and even mode. To isolate the losses added by the dissipative medium, since the Q-factor of the resonators in free space is usually high, the undamped resonant frequencies for two coupled coils can be sought by ignoring the internal resistance of the coil ($Z = j\omega L - 1/(j\omega C)$). The natural response of the system can be written as:

$$\begin{bmatrix} \tilde{Z} & j\omega\mathcal{M} \\ j\omega\mathcal{M} & \tilde{Z} \end{bmatrix} \begin{bmatrix} I_1 \\ I_2 \end{bmatrix} = \begin{bmatrix} 0 \\ 0 \end{bmatrix} \tag{S26}$$

$$\iff j\omega\left(L_0 + \Delta\mathcal{L}'\right) \begin{bmatrix} -j\dfrac{\Delta\mathcal{L}''}{L_0 + \Delta\mathcal{L}'} + 1 - \dfrac{\tilde{\omega_0}^2}{\omega^2} & \dfrac{\mathcal{M}}{L_0 + \Delta\mathcal{L}'} \\ \dfrac{\mathcal{M}}{L_0 + \Delta\mathcal{L}'} & -j\dfrac{\Delta\mathcal{L}''}{L_0 + \Delta\mathcal{L}'} + 1 - \dfrac{\tilde{\omega_0}^2}{\omega^2} \end{bmatrix} \begin{bmatrix} I_1 \\ I_2 \end{bmatrix} = \begin{bmatrix} 0 \\ 0 \end{bmatrix} \tag{S27}$$

where $\tilde{\omega_0} = 1/\sqrt{\left(L_0 + \Delta\mathcal{L}'\right) C_0}$ is the angular frequency of the coils inside the medium. Equation (S27) can be rearranged as:

$$\begin{bmatrix} -j\dfrac{\Delta\mathcal{L}''}{L_0 + \Delta\mathcal{L}'} + 1 & \dfrac{\mathcal{M}}{L_0 + \Delta\mathcal{L}'} \\ \dfrac{\mathcal{M}}{L_0 + \Delta\mathcal{L}'} & -j\dfrac{\Delta\mathcal{L}''}{L_0 + \Delta\mathcal{L}'} + 1 \end{bmatrix} \begin{bmatrix} I_1 \\ I_2 \end{bmatrix} = \frac{\tilde{\omega_0}^2}{\omega^2} \begin{bmatrix} I_1 \\ I_2 \end{bmatrix} \tag{S28}$$

Therefore, $\tilde{\omega_0}^2/\omega^2$ is the eigenvalue of the matrix on the left hand side of (S28). After simple

mathematical operations, the splitting frequency can be determined as:

$$f_{e,o} = \frac{\tilde{f}_0}{\sqrt{1 \pm \frac{\mathcal{M}}{L_0 + \Delta\mathcal{L}'} - j\frac{\Delta\mathcal{L}''}{L_0 + \Delta\mathcal{L}'}}} \tag{S29}$$

where $f_{e,o}$ denotes the upper and lower resonance after splitting. Because the Kirchhoff coefficients is generally complex, the eigenvalue $\tilde{\omega_0}^2/\omega^2$ and thereby, $f_{e,o}$ may be complex with the imaginary part representing the losses added by the dissipative surrounding medium. In practical strongly coupled magnetic resonators, the new splitting resonances are not much different, often within a factor of one tenth of the resonance in the isolated case. The complex coefficients therefore are taken the values at the resonance frequency of the coil in the conductive medium, $\mathcal{M} = \mathcal{M}(\tilde{\omega_0})$ and $\Delta\mathcal{L} = \Delta\mathcal{L}(\tilde{\omega_0})$. Finally, because the Q-factors of the coils in free space are high, the upper and lower resonant frequency is approximated by removing the imaginary part associated with the loss in the system, as shown in (2) in the manuscript.

2C. Current Distribution The antisymmetry of the current responses of two coupled circuits inside an attenuating medium indicates that resonant peaks undergo very different amounts of damping over the frequency range. From the equivalent circuit model, the voltage-current relationship in the transmitter can be rewritten as:

$$V_0 = \left[(Z_0 + j\omega\Delta\mathcal{L}) + \frac{(\omega\mathcal{M})^2}{Z_0 + j\omega\Delta\mathcal{L}}\right] I_1. \tag{S30}$$

In free space, (S30) reduces to:

$$V_0 = \left[Z_0 + \frac{(\omega M_0)^2}{Z_0}\right] I_1 \tag{S31}$$

Recall that $Z_0 = R_0 + jX_0$, (S31) can be rewritten as:

$$\begin{aligned} V_0 &= \left[R_0 + jX_0 + \frac{(\omega M_0)^2 R_0 - j(\omega M_0)^2 X_0}{R_0^2 + X_0^2}\right] I_1 \\ &= \left[R_0 + \frac{(\omega M_0)^2 R_0}{R_0^2 + X_0^2} + j\left(X_0 - \frac{(\omega M_0)^2 X_0}{R_0^2 + X_0^2}\right)\right] I_1 \end{aligned} \tag{S32}$$

As discussed above, the splitting resonance are not very separate. The additional reflected loss from the receiving coil $(\omega M_0)^2 R_0/(R_0^2 + X_0^2)$ is then approximately constant near the resonances. The well-known symmetry of the current response in coupled magnetic circuits arises as a result.

By contrast, due to the effects of the eddy currents, the complex self inductance arises while the complex mutual inductance becomes complex. Recall that: $\mathcal{M} = \mathcal{M}' - j\mathcal{M}''$ where $0 < \mathcal{M}', \mathcal{M}'' < M_0$, $\mathcal{L} = \mathcal{L}' - j\mathcal{L}''$ where $0 < \mathcal{L}', \mathcal{L}''$, and $Z_0 + j\omega\Delta\mathcal{L} = \tilde{R}_0 + j\tilde{X}_0$. Substituting the complex forms of the complex Kirchhoff coefficient and the new coil impedance into (S30), it results in:

$$V_0 = \left\{\left(\tilde{R}_0 + j\tilde{X}_0\right) + \frac{\left[\omega^2\left(\mathcal{M}'^2 - \mathcal{M}''^2\right) - j\,2\omega^2\mathcal{M}'\mathcal{M}''\right]\left(\tilde{R}_0 - j\tilde{X}_0\right)}{\tilde{R}_0^{\,2} + \tilde{X}_0^{\,2}}\right\} I_1 \tag{S33a}$$

$$\begin{aligned} &= \left\{\tilde{R}_0 + \frac{\left[\omega^2\left(\mathcal{M}'^2 - \mathcal{M}''^2\right)\tilde{R}_0 - 2\omega^2\mathcal{M}'\mathcal{M}''\tilde{X}_0\right]}{\tilde{R}_0^{\,2} + \tilde{X}_0^{\,2}}\right. \\ &\quad \left. + j\left[\tilde{X}_0 - \frac{2\omega^2\mathcal{M}'\mathcal{M}''\tilde{R}_0 - \omega^2\left(\mathcal{M}'^2 - \mathcal{M}''^2\right)\tilde{X}_0}{\tilde{R}_0^{\,2} + \tilde{X}_0^{\,2}}\right]\right\} I_1. \end{aligned} \tag{S33b}$$

where the term $\left[\omega^2\left(\mathcal{M}'^2 - \mathcal{M}''^2\right)\tilde{R}_0 - 2\omega^2\mathcal{M}'\mathcal{M}''\tilde{X}_0\right] / \left(\tilde{R}_0^{\,2} + \tilde{X}_0^{\,2}\right)$ is usually referred as the

loss from the feedback effect. Here, the numerator of the feedback loss is expressed:

$$\omega^2\left(\mathcal{M}'^2-\mathcal{M}''^2\right)\tilde{R}_0-2\omega^2\mathcal{M}'\mathcal{M}''\tilde{X}_0 \\ =\begin{cases}\omega^2\left(\mathcal{M}'^2-\mathcal{M}''^2\right)\tilde{R}_0+2\omega^2\mathcal{M}'\mathcal{M}''|\tilde{X}_0|, & \text{for } \omega<\tilde{\omega_0},\\ \omega^2\left(\mathcal{M}'^2-\mathcal{M}''^2\right)\tilde{R}_0, & \text{for } \omega=\tilde{\omega_0},\\ \omega^2\left(\mathcal{M}'^2-\mathcal{M}''^2\right)\tilde{R}_0-2\omega^2\mathcal{M}'\mathcal{M}''|\tilde{X}_0|, & \text{for } \omega>\tilde{\omega_0}.\end{cases} \tag{S34}$$

With the same argument for splitting resonances, the total energy loss due to the presence of the complex self inductance and the Ohmic loss, $\tilde{R}_0 = R_0 + \omega\Delta\mathcal{L}''$ might still be constant about the resonance. Nevertheless, from (S34), when the working frequency rises through the self resonance $\tilde{\omega_0}$, the reactance of the coils, $\tilde{X}_0$, crosses the zero line, changing from capacitive (negative value) to inductive (positive value). The progression of frequency indeed mitigates the feedback loss from the secondary coil. Therefore, the current in the transmitter exhibits an antisymmetric response with the lower resonance experiencing much higher damping than the upper resonance. Likewise, the current response in the receiver can be explained accordingly.

3 Experiment

3A. Dielectric Cavities, Supports, and Immersion Level Fig. S5a shows a diagram of a dielectric cavity used in the experiments. We place a measuring probe at the notch to access the near fields generated by the split ring resonators. Clearly, having a notch on the insulating cavities has a certain impact on the complex Kirchhoff coefficients because it causes more volume of the medium near the rings and in turn, larger eddy current effects. Also, the mathematical model is developed for a boundless medium background. However, the aqueous solution in the experiments is limited by the size of the container. To mimic the effect of an infinite medium, we thus study the effect of the immersion level of the coil in the tank, which is a key parameter to consider in designing the experimental framework. The immersion level, i_v, refers to the distance from the edge of the insulating cavity to the medium surface, as shown in Fig S5b. The region above the medium is vacuum.

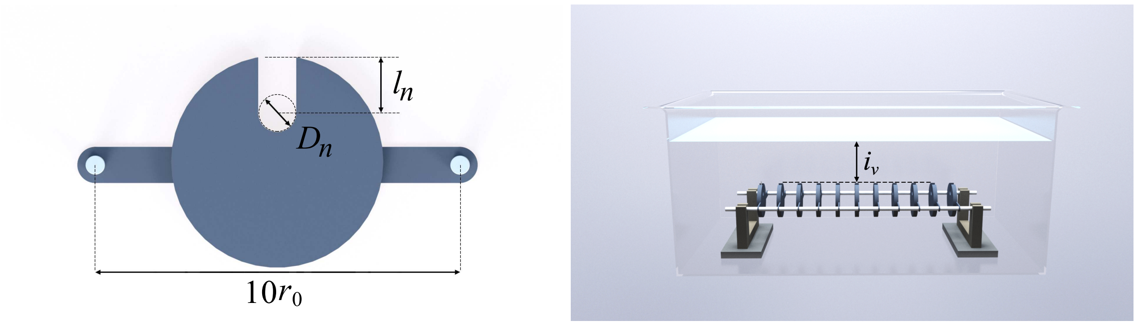

(a) (b)

Figure S5: (a) Diagram of the dielectric cavity providing the insulation for the split ring resonators. (b) Vertical immersion level of the split ring resonators in the experiment.

The combination effects of the notch and the immersion levels are simulated with CST EMS. Fig. S6 shows the normalised modulus and phase of the complex coefficients as a function of the immersion levels with the actual design for insulating cavities. We perform the simulations at the resonance frequency $f_0 = 46$ MHz. The results are normalised to the simulated values for the configuration where the medium background is infinite and the insulators are perfectly cylindrical in shape.

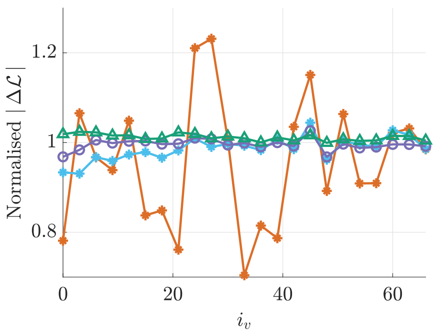

(a) The real part of $\Delta\mathcal{L}$

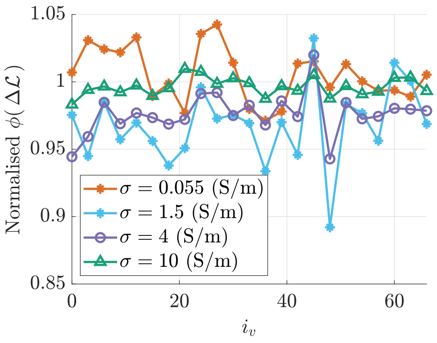

(b) The imaginary part of $\Delta\mathcal{L}$

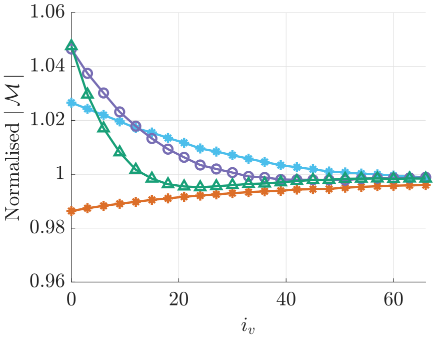

(c) The real part of $\mathcal{M}$

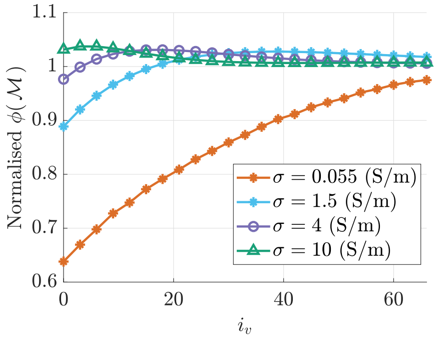

(d) The imaginary part of $\mathcal{M}$

Figure S6: Normalised complex Kirhhoff coefficients $\Delta\mathcal{L}$ and $\mathcal{M}$ as a function of the immersion level. The vertical distance between the two coils is $d = 30$ mm while the horizontal displacement is $\Delta d = 0$ mm. The dissipative medium has relative permittivity of $\epsilon_r = 78$, relative permeability of $\mu_r = 1$, and variable conductivity $\sigma = 0.055$, 1.5, 4, and 10 S/m. CST EMS simulation results for the insulating cavities shown in Fig. S5a are normalised to that of the case when the surrounding medium is infinite and the insulators are perfectly cylindrical in shape.

In conjunction with the notch, the immersion depth modifies both complex coefficients. However, when the coils are submerged with a depth of $i_v \geq 60$ mm, it plays a less important

role as the mean absolute percentage errors in both the modulus and phase are less than 3% compared to the reference values for both complex Kirchhoff terms. The immersion level therefore should be larger than 60 mm for this particular configuration to corroborate the accuracy of the analytical model. As a result, the U-shaped supports used to carry the rods interconnecting the dielectric insulator have a height of $h_c = 100$ mm to satisfy the immersion level conditions.

Finally, the insulating cavities and the U-shaped supports are realized by using 3D printing with acrylonitrile butadiene styrene (ABS) filament, see Fig. S7. To prevent water leakage, the dielectric cavities are sealed with silicone adhesive sealant (LOCTITE SI 595).

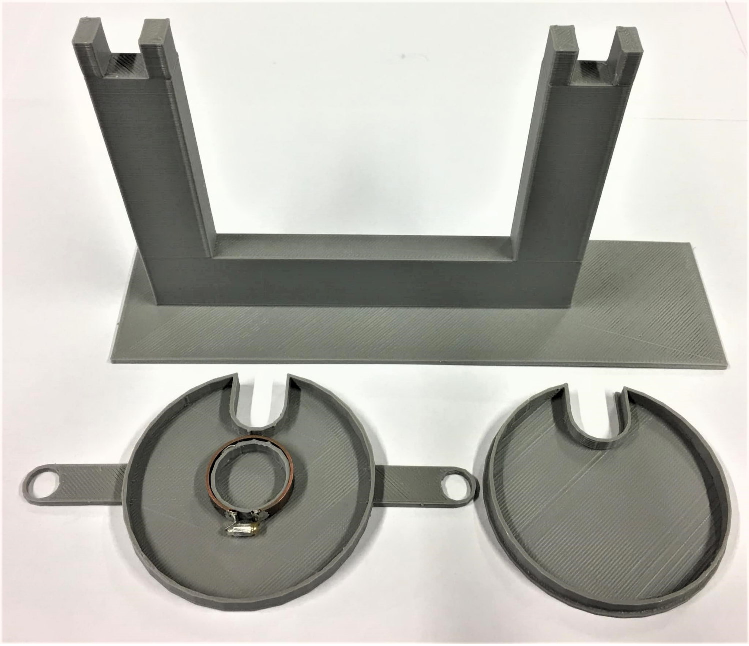

Figure S7: A 3D printed (top) U-shaped cantilevers, the (bottom right) body and (bottom left) cap of an insulating cylindrical cavity with a split ring resonator inside.

Resonator Characterization In this part, we report the resonator characterization and the effects of the insulating boxes. When insulating the split rings by placing them inside non-magnetic cavities, the cavity material having a dielectric constant different from that of vacuum may introduce a small capacitance to the circuits. Predictably, it leads to a small decrease in the self resonance of the rings. Fig. S8 shows the transmission-type experimental apparatus in order to measure the Q-factor and resonant frequency of each coil with and without dielectric cavities. This method is widely employed because of its better accuracy compared to other methods, given that the two measuring prboes are in loosly coupled regime[8,9]. Here, we placed a pair of weakly-coupling probes in close proximity to the coils under test, which were supported by a Balsa-wood fixture, and recorded the transmission coefficient S_{21}.

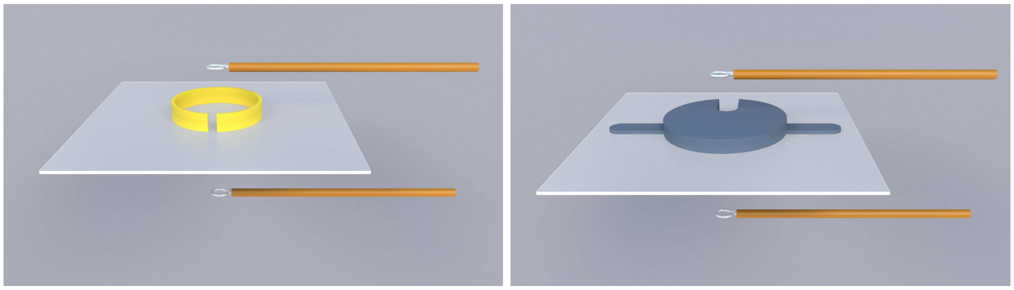

(a) (b)

Figure S8: The measurement set-up to measure the quality factor of the fabricated split ring resonators (a) alone and (b) in combination with their corresponding insulating cavities.

To increase the accuracy, the complex transmission coeffcient S_{21} is fit to a Lorentzian curve

using a nonlinear least-squares fit[9]:

$$|S_{21}(f)|^2 = A_1 + A_2 f + \frac{|S_{max}| + A_3 f}{\sqrt{1 + 4\left(\frac{f - f_0}{\Delta f_{Lorent}}\right)^2}} \tag{S35}$$

where the fitting parameters are as follows: f_0 is the resonant frequency, Δf_{Lorent} is the bandwidth, $|S_{max}|$ is the maximum magnitude, A_1 is the constant background, A_2 is the slope on the background, and A_3 is the skew. The Q-factor is then determined using the fit parameters f_0 and Δf_{Lorent}:

$$Q = \frac{f_0}{\Delta f_{Lorent}} \tag{S36}$$

A large number of coils are manufactured and tested. In this document, we only detailed the resonant frequency and Q-factor of the coils that were selected to build MI waveguides, as shown in Fig. S9.

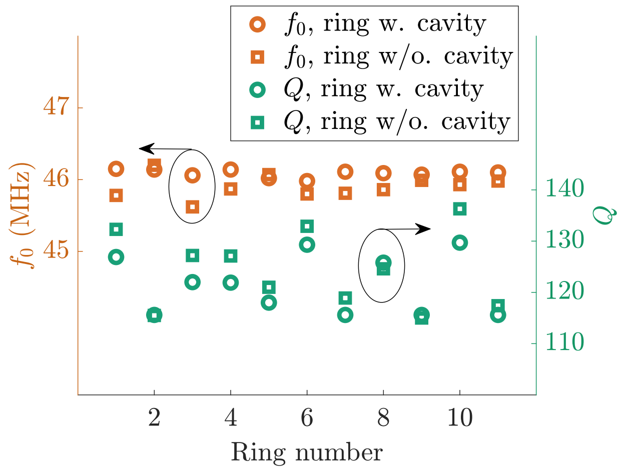

Figure S9: Resonant frequency, f_0, and Q-factors, Q, of split ring resonators with and without their corresponding insulators calculated from the Lorentzian fit.

Experimental Apparatus Fig. S10 shows photographs of the actual experiment apparatus in the laboratory.

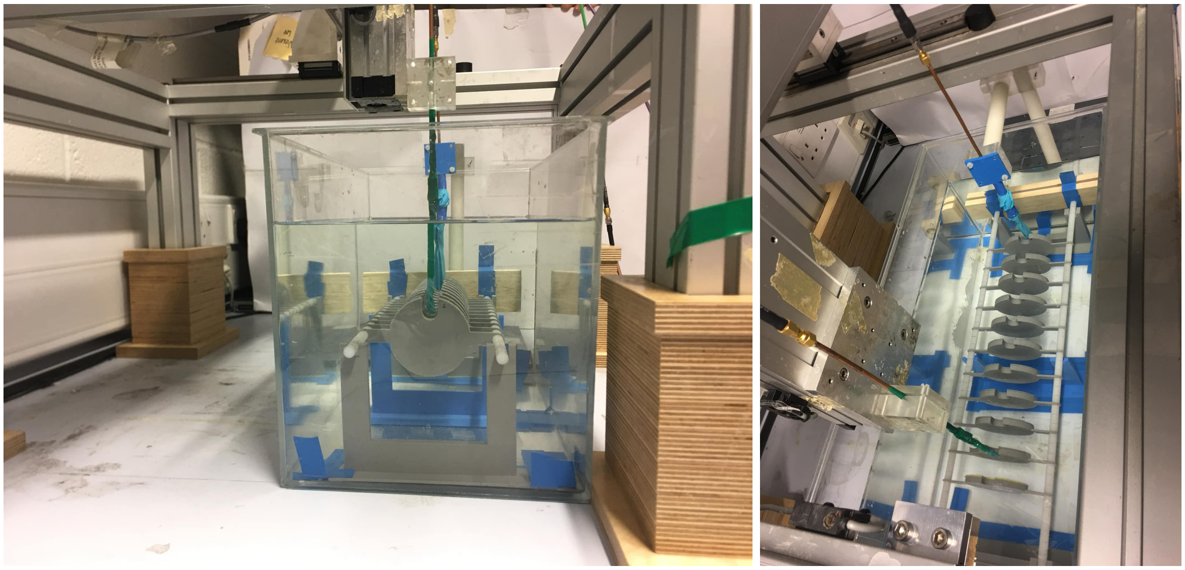

(a) (b)

Figure S10: Laboratory measurement of an MI waveguide consisting of 11 elements with the distance between two adjacent elements of $d = 30$ mm submerged in an aqueous solution of sodium chloride with suitable conductivity. (a) Side view, and (b) top view.

Experimental Results Fig. S11 presents the magnitude of the measured signals at 46 MHz as a function of the element position. The -100 dB line (light grey) indicates the noise floor in the measurement.

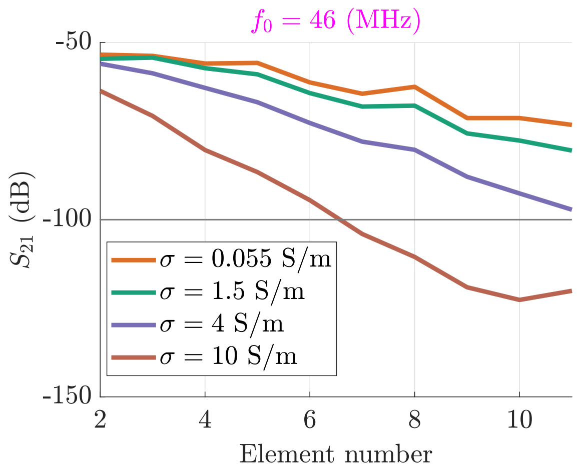

Figure S11: Magnitude of the measured signals at 46 MHz as a function of the element position.

1. Griffiths, D. *Introduction to Electrodynamics* (Pearson Education, 2014), 4th edn.

2. Desjardins, D., Krause, T., Tetervak, A. & Clapham, L. Concerning the derivation of exact solutions to inductive circuit problems for eddy current testing. *NDT & E Int.* **68**, 128–135 (2014).

3. Conway, J. T. Mutual inductance for an explicitly finite number of turns. *Prog. Electromagn. Res. B* **28**, 273–287 (2011).

4. Conway, J. T. Inductance calculations for noncoaxial coils using bessel functions. *IEEE Trans. Magn.* **43**, 1023–1034 (2007).

5. Chu, S., Vallecchi, A., Stevens, C. J. & Shamonina, E. Fields and coupling between coils embedded in conductive environments. *EPJ Applied Metamaterials* **5** (2018).

6. Chu, S. *Near-fields in attenuating media*. Ph.D. thesis, University of Oxford (2020).

7. Ahn, D. & Hong, S. A study on magnetic field repeater in wireless power transfer. *IEEE Trans. Ind. Electron.* **60**, 360–371 (2012).

8. Kajfez, D., Chebolu, S., Abdul-Gaffoor, M. & Kishk, A. Uncertainty analysis of the transmission-type measurement of q-factor. *IEEE Trans. Microw. Theory Tech.* **47**, 367–371 (1999).

9. Petersan, P. J. & Anlage, S. M. Measurement of resonant frequency and quality factor of microwave resonators: Comparison of methods. *J. Appl. Phys.* **84**, 3392–3402 (1998).
